# Event-Related Potentials and executive control deficits in major depression: evidence from the Attention Network Test

**DOI:** 10.3389/fnsys.2025.1674124

**Published:** 2026-01-16

**Authors:** Almira Kustubayeva, Manzura Zholdassova, Altyngul Kamzanova, Zabira Madaliyeva, Aigul Suleimenova, Sultangali Nessipbayev, Gulnur Borbassova, Diana Arman, Erik Nelson, Gerald Matthews

**Affiliations:** 1 Al-Farabi Kazakh National University, Almaty, Kazakhstan; 2 Departments of Biophysics, Biomedicine, and Neuroscience, Al-Farabi Kazakh National University, Almaty, Kazakhstan; 3 Department of Psychology, Al-Farabi Kazakh National University, Almaty, Kazakhstan; 4 Republican Scientific and Practical Center for Mental Health, Almaty, Kazakhstan; 5 Keruen-Medicus, Almaty, Kazakhstan; 6 School of Information Technology and Engineering, Kazakh-British Technical University, Almaty, Kazakhstan; 7 Department of Psychiatry and Behavioral Neuroscience, College of Medicine, University of Cincinnati, Cincinnati, OH, United States; 8 Department of Psychology, George Mason University, Fairfax, VA, United States

**Keywords:** alerting, Attention Network Test, biomarkers, Event-Related Potentials, executive control, major depressive disorder

## Abstract

**Objective:**

Behavioral and neurological studies suggest that major depressive disorder (MDD) is associated with pervasive deficits in executive control of attention. Research using Event-Related Potentials (ERPs) to investigate attentional impairments in depression has provided mixed results. The current study aimed to clarify abnormalities in ERPs associated with depression through use of the Attention Network Test (ANT) which assesses efficiency of three fundamental brain networks: executive control, alerting, and orienting.

**Methods:**

Participants were 93 volunteers. We compared ERP amplitudes in healthy, subsyndromal depression, and MDD groups (31 participants per group) during performance of an extended-duration version of the ANT.

**Results:**

Both N100 and P300 ERP amplitudes were generally lower in the MDD group across central-parietal and posterior sites, with medium-to-large effect sizes. There were also significant effects of depression on the ANT indices for executive control and alerting. Further analyses showed that some abnormalities in ERPs were seen in the subsyndromal group and that depression effects were stable across time, despite vigilance decrement.

**Conclusion:**

Neurocognitive deficits in depression may relate to depletion of a general attentional resource.

## Highlights

- Utilizes Attention Network Test (ANT) and Event-Related Potentials (ERPs) to investigate executive control deficits in major depression.
- Demonstrates reduced amplitudes of both earlier and later ERPs in depressed patients, and, to some extent, in individuals with subsyndromal depression.
- Identifies ERP metrics that may be useful as biomarkers in clinical practice.

## 1 Introduction

Depression is associated with deficits in multiple cognitive functions, including learning and memory, attention and concentration, executive function, and information processing speed (Ahern and Semkovska, 2017; Liu et al., 2019; Zuckerman et al., 2018). Depression is also associated with cognitive biases in processing affectively valent stimuli, such as preferential selective attention and recall for negative material (LeMoult and Gotlib, 2019).

There are three principal hypotheses for the role of cognitive dysfunction in the etiology and maintenance of clinical depression that are referred to as state, trait, and scar models (Ahern and Semkovska, 2017; Clark et al., 1994). Cognitive dysfunction may reflect the immediate impacts of depression on performance (state), stable cognitive impairments that precede onset of the illness (trait) or enduring neurocognitive damaging resulting from a depressive episode (scar). All three hypotheses may have some validity, depending on the cognitive function concerned (Ahern and Semkovska, 2017).

### 1.1 Executive function deficits in depression

Cognitive deficits in depression might reflect some global deficit such as lack of attentional capacity or deficient task-directed effort (Wang et al., 2020). However, deficiencies in attention may also result from specific processes or brain networks (Nuño et al., 2021). There has been a recent focus on deficits in executive function or cognitive control as a source of multiple performance impairments in depression (Kustubayeva et al., 2020; Kustubayeva A. M. et al., 2022). Executive function refers to a set of cognitive control processes such as working memory updating, selective inhibition of task-irrelevant material, and set-shifting that collectively support voluntary regulation of information-processing (Miyake et al., 2000). Executive function deficits are prevalent in both major depressive disorder (MDD) and subclinical depression (Dotson et al., 2020; Nuño et al., 2021). Such deficits may contribute to dysfunctional emotion regulation in depression (Joormann and Vanderlind, 2014; Kustubayeva et al., 2013).

### 1.2 Event-Related Potentials and attention in depressed individuals

Neuropsychological studies can identify specific mechanisms for cognitive deficits associated with clinical disorders (Pultsina et al., 2022). Studies of depression have used Event-Related Potentials (ERPs) measured through electroencephalography (EEG). The various ERP waveforms index different cognitive processes ranging from early stimulus encoding (N100) to later higher-level processes such as working memory updating (P300). Studies of depression and the amplitude of early waves (N100, P200, N200) have provided inconsistent results (Bruun et al., 2021; De Aguiar Neto and Rosa, 2019). Compared to healthy controls, patients with depression have been reported to show both higher and lower amplitudes of early ERPs, with several studies showing no effect of depression.

Findings regarding depression and amplitudes of later waves including P300 and the Late Positive Potential (LPP) are also mixed. Ilardi et al. (2007) concluded from their review of studies published between 1985 and 2000 that P300 response in depression was consistently attenuated across a variety of stimulus types and sensory modalities. More recent reviews have drawn more qualified conclusions. Three reviews (Bruun et al., 2021; De Aguiar Neto and Rosa, 2019; Greco et al., 2021) identified instances of P300 attenuation in depressed patients but also highlighted variability in findings across studies. Kangas et al. (2022) concluded that depression influenced the P3b component of P300, indexing conscious attention and memory updating, but not the P3a component associated with bottom-up capture of attention and orienting.

The capacity of ERP metrics to discriminate depressed individuals is dependent on the stimuli and task used (Greco et al., 2021), and previous studies may not have systematically sampled tasks to represent processes supporting stimulus encoding and early stages of selective attention. Much research has used the oddball task, which simply requires recognition of a low-probability stimulus. A recent meta-analysis (Arıkan et al., 2024) reported an effect size of 0.49 for the difference in P300 amplitude between major depression patients and healthy controls. However, use of the oddball task in preference to other attentional task paradigms may be a limitation of ERP and depression research (Nandrino et al., 1996). Additional methodological issues including psychiatric comorbidity, medication status, and variation in subtype and severity of depression may also contribute to inconsistency in findings (Greimel et al., 2022).

### 1.3 The Attention Network Test as a research tool

Petersen and Posner’s (2012) account of multiple attention networks discriminated three brain systems on the basis of converging behavioral and neuroimaging evidence. The executive control network is responsible for top-down regulation of attention to task-relevant information while suppressing irrelevant or conflicting information. It is supported by brain structures including anterior cingulate cortex (ACC) and areas of prefrontal cortex. The alerting network maintains attention following arrival of a warning of cue stimulus, supported by ascending noradrenergic arousal pathways and parietal and frontal areas. The orienting network focuses attention on a specific input pathway, such as spatial location. Orienting is based on both ventral and dorsal frontal-parietal pathways.

The Attention Network Test (ANT: Fan et al., 2002) measures the impact of each network on response speed during attentional task performance. Executive control is operationalized as resistance to incongruent flanker effects on target discrimination, alertness as the benefit of a warning cue prior to discrimination, and orienting as the benefit of spatial cue. Indices for the three networks are calculated as difference scores for reaction time (RT) with the relevant flanker/cue present versus RT in a control condition. Research has confirmed that the three indices are largely independent of one another, although dependencies that reflect interactions between the three networks may occur (Fan et al., 2002, 2007). Several variants of the original ANT have also been developed (De Souza Almeida et al., 2021; Roca et al., 2011).

Researchers have utilized the ANT to address a variety of issues (Klein, 2022). These include its application to explore the nature of attentional dysfunction in various clinical conditions including schizophrenia (Spagna et al., 2018), generalized anxiety disorder (Najmi et al., 2014), and attention deficit/hyperactivity disorder (ADHD: Arora et al., 2020). Eleven studies were included in a meta-analytic review of depression effects on the ANT (Sinha et al., 2022). The analysis showed a significant effect of depression on the executive control index. On average, the median value for the index was 19 ms greater in depressed compared to control participants, indicating weaker control. Group differences for alerting and orienting were close to zero and non-significant. However, samples were quite heterogeneous, and not all studies showed the typical depression effect. The specificity of the depression effect to ANT executive control is consistent with behavioral evidence from other control tasks (Dotson et al., 2020; Nuño et al., 2021) and with ERP studies (Kangas et al., 2022). Studies using fMRI also broadly suggest executive impairment (Elliott et al., 2002; Kustubayeva et al., 2023; Kustubayeva A. M. et al., 2022). Impairment in MDD may be associated with both reduced and increased activation in frontal areas depending on the task performed and whether performance is maintained (Pilmeyer et al., 2022; Wang et al., 2015). Increased activity in anterior cingulate and prefrontal cortex indicates efforts to compensate for neural inefficiency (Pilmeyer et al., 2022). In addition, increased activation in these and other areas may reflect default network activity associated with rumination and abnormality in functional connectivity in the executive network (Hamilton et al., 2011; Sendi et al., 2020).

Event-Related Potential studies can complement behavioral data on network function. In non-clinical samples, the ANT has been used to investigate ERPs during attentional task performance in several studies (Chen et al., 2024; Galvao-Carmona et al., 2014; Kaufman et al., 2016; Kustubayeva A. et al., 2022; Neuhaus et al., 2010). The characteristic finding is that alerting and orienting are associated with N100 and other early wave responses in posterior sites, consistent with the role of these networks in early stages of stimulus encoding and selective attention (Petersen and Posner, 2012). ANT executive control typically relates to parietal P300; i.e., incongruent flankers produce a smaller-amplitude P300. Effects may differ at frontal sites: Kustubayeva A. et al. (2022) found that alerting and orienting were associated with Fz frontal response but executive control was not. There is little evidence on depression effects on ERP responses to the ANT. Yang and Xiang (2019) found a significant effect of depression on the conflict-sustained potential (SP) 500–650 ms after stimulus onset, indicating an impairment in executive control.

### 1.4 Aims and hypotheses

The principal aim of the study was to test for effects of depression on earlier (N100) and later (P300) ERP responses during performance of the ANT. Data were obtained from healthy controls, MDD patients, and a subsyndromal depression group who did not meet clinical criteria for an MDD diagnosis but exhibited depressive symptomatology. Previous behavioral studies (Dotson et al., 2020; Nuño et al., 2021) suggest that depressed individuals exhibit deficits in executive function, including studies using the ANT executive control measure (Sinha et al., 2022), control of attention. Parietal/central-parietal P300 amplitude is sensitive to executive network activation (Kustubayeva A. et al., 2022; Neuhaus et al., 2010). That is, P300 amplitude is smaller with incongruent relative to congruent flankers. We hypothesized that weaker executive control in depressed individuals would be expressed as (1) higher values of the behavioral executive control index and (2) attenuation of the parietal P300 flanker incongruence effect, relative to healthy controls. We investigated effects on N100, on alerting and orienting networks, and on frontal ERPs on an exploratory basis. In general, previous depression research does not support predicting effects beyond parietal P300 and executive control. However, the association between depression and prefrontal impairment implies that depression effects on frontal ERPs especially merit further investigation.

We also aimed to investigate two further issues: depression effects on sustaining attention over time, and ERP characteristics of a subsyndromal depression group. The ANT has been used to investigate sustained attention, either through introducing an additional index for vigilance (Roca et al., 2011) or through testing for temporal decline in performance. In studies using a long-duration version of the ANT (>60 min), we found that temporal deficits in performance were inconsistent across studies, and, when found, appeared to be strongest in a no-cue control condition (Kustubayeva A. et al., 2022; Zholdassova et al., 2021). However, ERP data provided a clearer picture of temporal declines with evidence for decreased N100 alerting response at parietal-occipital sites, as well as frontal declines in both N100 and P300. Temporal decline in sustained attention is attributed to cognitive fatigue and depletion of attentional resources (Matthews et al., 2017). Individual differences in fatigue correlate with impairments in executive control (Matthews and Zeidner, 2012). Thus, we hypothesized that depressed individuals would show decreasing executive control over time, as measured by the relevant ERP index. An effect of this type might identify a state rather than a trait effect of depression.

Current research has not determined conclusively whether ANT executive control effects in depression reflect trait, state, or scar effects (Ahern and Semkovska, 2017). To distinguish possible trait and state effects, we tested both a group diagnosed for the first time with MDD and a subsyndromal group comprised of individuals with high scores on a depression trait measure who did not meet the criteria for a diagnosis of MDD. Longitudinal studies have shown that subsyndromal (or subthreshold) individuals are at increased risk of future development of MDD (Lee et al., 2019; Pietrzak et al., 2013). Subsyndromal depression is also associated with behavioral and neurological deficits in attention that may generate vulnerability to clinical disorder (Hwang et al., 2015; Li et al., 2024). Thus, we hypothesized that a trait effect on executive control would appear in both groups, though perhaps more strongly in the clinical group. A state effect would be limited to the MDD group alone. Evidence for an association between subclinical depression and executive impairment in people with no clinical history (e.g., Marchetti et al., 2018) supports the hypothesis that both the MDD group and subsyndromal group would differ from healthy controls in behavioral and ERP measures of executive control.

## 2 Materials and methods

### 2.1 Participants

Participants were 93 volunteers selected to form three groups: (1) healthy group (HG) with a mean age of 24.5, SD = 6.5; (2) subsyndromal depression group (SG) with a mean age of 23.9, SD = 8.2; (3) major depression group (DG) with a mean age of 24.2, SD = 7.8. There were 7 males and 24 females in each group. The Ethics Committee of the Faculty of Medicine and Health Care of the Al-Farabi Kazakh National University provided approval to conduct the study. A written consent form was obtained from all participants. Inclusion criteria were: no previous history of psychiatric or neurological illness, normal or corrected vision, right-handed, first time diagnosed with MDD (for DG) and before receiving any antidepressant treatment. People who reported substance abuse in the psychiatric interview were excluded from the sample.

Participants completed the self-report version of the 30-item Inventory of Depressive Symptomatology (IDS: Rush et al., 1996). Subjects who had an IDS score higher than 30 were then interviewed by a clinical psychologist and by a psychiatrist to establish whether a current major depressive episode (MDE) was present based on the International Statistical Classification of Diseases and Related Health Problems (ICD-10). Participants assigned to the SG met the same criterion of IDS score greater than 30 but did not meet the criteria for a major depressive episode based on the clinical interview. Mean (and SDs) on the IDS were 16.6 (7.9) for HG, 35.8 (7.7) for SG and 37.5 (11.3) for DG. Between-groups *t*-tests, with the Bonferroni correction applied, showed significant (*p* < 0.01) mean differences between the healthy group and both subsyndromal and depressed groups (*p* < 0.01), but the subsyndromal and depressed groups did not differ significantly from one another.

All participants were required to abstain from drinks and foods containing caffeine prior to the study. Data were collected during the first half of day (between 08.00 and 13.00 h).

### 2.2 Questionnaires

Questionnaires were administered in the Kazakh language. The IDS (self-report version: Rush et al., 1996) assesses overall severity of a range of depressive symptoms including emotional, cognitive, somatic and other symptoms, developed to provide a more sensitive measure of depression than other scales. The Dundee Stress State Questionnaire (DSSQ: Matthews et al., 2002) was developed to assess multiple dimensions of acute stress response to task performance. This study utilized DSSQ scales for mood only: energetic arousal, tense arousal, hedonic tone (pleasantness of mood), and anger/frustration. Validation studies have shown that scales are appropriately sensitive to a variety of task and environmental stressors (Matthews, 2021). Matthews and Southall (1991) found that depressed subjects were high in tense arousal but low in energetic arousal and hedonic tone, relative to healthy controls.

### 2.3 Attention Network Test (ANT)

Participants performed the modified version of ANT task (Fan et al., 2002; Kustubayeva A. et al., 2022; Zholdassova et al., 2021) programmed in E-Prime 2.0 software (Psychology Software Tools, Pittsburgh, PA). It was modified by extending the duration to approximately 70 min for use in sustained attention research. The task consisted of nine blocks of 96 trials. Cue type and flanker type were manipulated across trials as specified by Fan et al. (2002) to assess each of the three Petersen and Posner (2012) attentional networks. All stimulus characteristics were systematically counterbalanced in a predetermined pseudorandom sequence. Response time to the target in milliseconds and accuracy were logged for each trial.

The stimuli were displayed in black against a white background, with the task screen positioned 65 cm from the participant’s eyes (see Kustubayeva A. et al., 2022, for full details). Following the presentation of an initial fixation cross and a cue stimulus, a central arrow stimulus (the target) emerged on the screen. Depending on the arrow’s direction, participants were required to respond promptly and accurately using left and right response keys. Each trial utilized one of four cue categories: no cue, double cue, central cue, or spatial cue (above or below the fixation cross). Additionally, there were three flanker categories: congruent, incongruent, or neutral.

Indices of executive control, alerting, and orienting were calculated from Fan et al.’s (2002) formulae: (1) Executive Control = mean RT for incongruent flanking trials - mean RT for congruent flanking trials; (2) Alerting = mean RT for no-cue trials - mean RT for double-cue trials; (3) Orienting = mean RT of center cue trials - mean RT of spatial cue trials.

### 2.4 EEG recording

Neuron-Spectrum_4 system (Neurosoft Ltd., Ivanovo, Russia) was used for EEG recording based on the 10%–20% international system from frontal, temporal, parietal, occipital, and central electrodes (FPz, F3, F4, F7, F8, Fz, FCz, C3, C4, Cz, CPz, P3, P4, Pz, O1, O2, Oz), with an indifferent ear electrode. Recording occurred in various situations: open eyes (1 min), closed eyes (1 min), and ANT task performance (70 min). Horizontal and vertical electro-oculograms (HEOG, VEOG) were recorded. The sampling rate during recording was set at 256 Hz, and impedance remained below 5 kOhm throughout the experiment. The E-Prime ANT task program was synchronized with EEG recording and sent markers at the onset of task stimuli, including the start, different cue conditions (no cue, double cue, center cue, spatial cue - up and down), and the target/flankers (congruent, incongruent, neutral).

### 2.5 EEG pre-processing

The EEG/ERPlab toolbox (Delorme and Makeig, 2004; Lopez-Calderon and Luck, 2014) was used for data preprocessing and the analysis of ERP parameters for electrodes at central and parietal-occipital locations (Fz, Cz, Pz, FPz, FCz, CPz, P3, P4, O1, O2). The preprocessing steps encompassed DC correction, bandpass filtering (0.1–30 Hz), epoching, baseline correction, artifact removal through the ICA (Independent Component Analysis) runica.m (Bell and Sejnowski, 1995) algorithm, artifact rejection (threshold: 75 uV). Measurements of N100 and P300 amplitude and latency were obtained from artifact-free EEG epochs within the time window of −700 ms pre-stimulus to +700 ms post-stimulus, specifically for congruent, neutral, and incongruent flankers, with pre-stimulus baseline correction in the interval [−700 to −400]. The epoching time range of [−700 to 700] was selected based on the methodology outlined in the previous work (Kustubayeva A. et al., 2022).

### 2.6 Statistical analysis

Statistical analysis of behavioral data (mean RT) and ERP amplitude and latency was done using the SPSS 25.0 package (IBM Corp, 2017). E-DataAid provided calculations of mean RT. Data were averaged across trial blocks 1–3, 4–6 and 7–9 to define three temporal stages of the task.

Event-Related Potential amplitudes were calculated for responses to the initial cue and to the subsequent target stimulus. N100_cue was calculated only for analyses of networks activated by cues (alerting and orienting). N100 amplitude was calculated for both cue (N100_cue) and target (N100_tar) and P300 for target only (P300_tar). Based on inspection of voltage plots, the following intervals were chosen to capture ERP amplitude: for N100_cue wave - −150 to −250 ms; for N100_tar wave - 225 – 300 ms; for P300_tar wave from 250 to 600 ms.

Two sets of analyses were run for each network. First, we ran “combined-electrode” analyses that averaged amplitudes from multiple electrodes at sites associated with the network. Based on previous studies (Kaufman et al., 2016; Kustubayeva A. et al., 2022; Neuhaus et al., 2010), the executive control network response was averaged across Cz and CPz channels, and the alerting and orienting responses were averaged across Pz, P3, P4, O1, and O2 channels. The executive control network is said to primarily influence P300 whereas alerting and orienting are expressed in the N100 (Neuhaus et al., 2010). We report both waves for each network here (see Kustubayeva A. et al., 2022).

Second, ERPs at Fz were analyzed to investigate changes in frontal activation previously implicated in vigilance decrement (Langner and Eickhoff, 2013; Nelson et al., 2014). For comparison with behavioral data, ERP amplitude-based network indices were calculated, using the combined-electrode measures: (1) Executive Control/inhibition = ERP amplitude for congruent flanking trials - ERP amplitude for incongruent flanking trials; (2) Alerting = ERP amplitude for double-cue trials - ERP amplitude for no-cue trials; (3) Orienting = ERP amplitude of spatial cue trials - ERP amplitude of center cue trials.

Behavioral and ERP data were analyzed using a 2 × 3 × 3 (flanker/cue type × group × stage) repeated-measures ANOVA design. Data for each network were analyzed separately. The breakdown of ERP data by stage is given in Supplementary Appendix 3; significant stage effects are described in the Section “3 Results.” To illustrate graphically the major effects of cue type and group, we also present voltage plots and 2-D topographic maps.

The ANOVAs aimed to test *a priori* hypotheses rather than to perform exploratory analyses. In general, the ANOVAs either replicated well-substantiated effects of the experimental factors on RT (Fan et al., 2002) and ERPs (Neumann et al., 2010) or tested hypotheses justified from related work such as the impact of depression on executive functioning (Nuño et al., 2021) and vigilance effects on ERPs (Kustubayeva A. et al., 2022). However, there may be a concern that the multiple tests may generate false positive findings associated with Type 1 error. We report both uncorrected *p*-values for the ANOVA *F*-tests and *p*-values adjusted using the Benjamini-Hochberg correction (Benjamini and Hochberg, 1995), denoted by *p*_*BH*_. The correction aims to limit the False Discovery Rate to 5% and it is considered to provide an appropriate balance between Type 1 and Type 2 errors (Thissen et al., 2002). Significant findings that fail to reach significance following the correction should be regarded as tentative.

## 3 Results

### 3.1 DSSQ: effects of group and task performance

Group differences in affective states pre- and post-task performance were assessed using the DSSQ mood scales. These analyses also tested whether the groups reacted differentially to performing a long-duration task given that state changes during the task might produce loss of vigilance (Matthews et al., 2017). All state scales were analyzed with 3 × 2 (group × pre/post) mixed-model ANOVAs with repeated measures on pre/post. Cell means are given in Table 1. There were significant main effects of group on energetic arousal (*F*(2,90) = 12.109, *p* < 0.01, *p*_*BH*_ < 0.01, η_*p*_^2^ = 0.212), tense arousal (*F*(2,90) = 4.544, *p* < 0.05, *p*_*BH*_ < 0.05, η_*p*_^2^ = 0.092), hedonic tone (*F*(2,90) = 15.541, *p* < 0.01, *p*_*BH*_ < 0.01, η_*p*_^2^ = 0.256), and anger/frustration (*F*(2,90) = 10.246, *p* < 0.01, *p*_*BH*_ < 0.01, η_*p*_^2^ = 0.185). Compared to HG, DG was higher in tension and anger, and lower in energy and hedonic tone, before and after performance. Scores for SG tended to be intermediate between HG and DG scores. The pre/post factor indexes state change following task performance. Main effects of pre/post were associated with decreasing hedonic tone (*F*(1,90) = 5.200, *p* < 0.050, *p*_*BH*_ < 0.01, η_*p*_^2^ = 0.055), and increasing anger frustration (*F*(1,90) = 7.077, *p* < 0.01, *p*_*BH*_ < 0.01, η_*p*_^2^ = 0.073) but there was no significant change in energetic arousal and tense arousal. The group × pre/post interaction was significant for tense arousal (*F*(2,90) = 3.117, *p* = 0.049, *p*_*BH*_ < 0.10, η_*p*_^2^ = 0.065), hedonic tone (*F*(2,90) = 10.695, *p* < 0.01, *p*_*BH*_ < 0.01, η_*p*_^2^ = 0.192), and anger/frustration (*F*(2,90) = 14.785, *p* < 0.01, *p*_*BH*_ < 0.01, η_*p*_^2^ = 0.247). Hedonic tone declined for HG, but improved for SG, and, to a lesser extent, for DG. Somewhat similarly, anger/frustration increased over time for HG, declined for SG, and increased slightly for DG.

**TABLE 1:** Means (and SDs) for DSSQ mood scales pre- and post-task, in three groups.

DSSQ mood scales	Healthy group (HG)				Subsyndromal group (SG)				Depressed group (DG)			
Scale	Pre-task		Post-task		Pre-task		Post-task		Pre-task		Post-task	
Energetic arousal	24.9	(2.9)	23.9	(4.2)	23.0	(3.9)	22.5	(3.7)	20.5	(4.6)	19.8	(4.3)
Tense arousal	13.1	(3.4)	14.8	(4.6)	16.5	(4.6)	15.5	(3.7)	17.1	(5.3)	16.7	(5.5)
Hedonic tone	28.3	(2.9)	26.8	(4.1)	22.4	(4.6)	25.9	(4.6)	22.0	(4.4)	23.0	(4.3)
Anger frustration	6.6	(1.4)	7.4	(2.3)	12.1	(5.4)	7.6	(2.5)	9.4	(3.5)	9.6	(3.8)

### 3.2 Behavioral data

**FIGURE 1:**
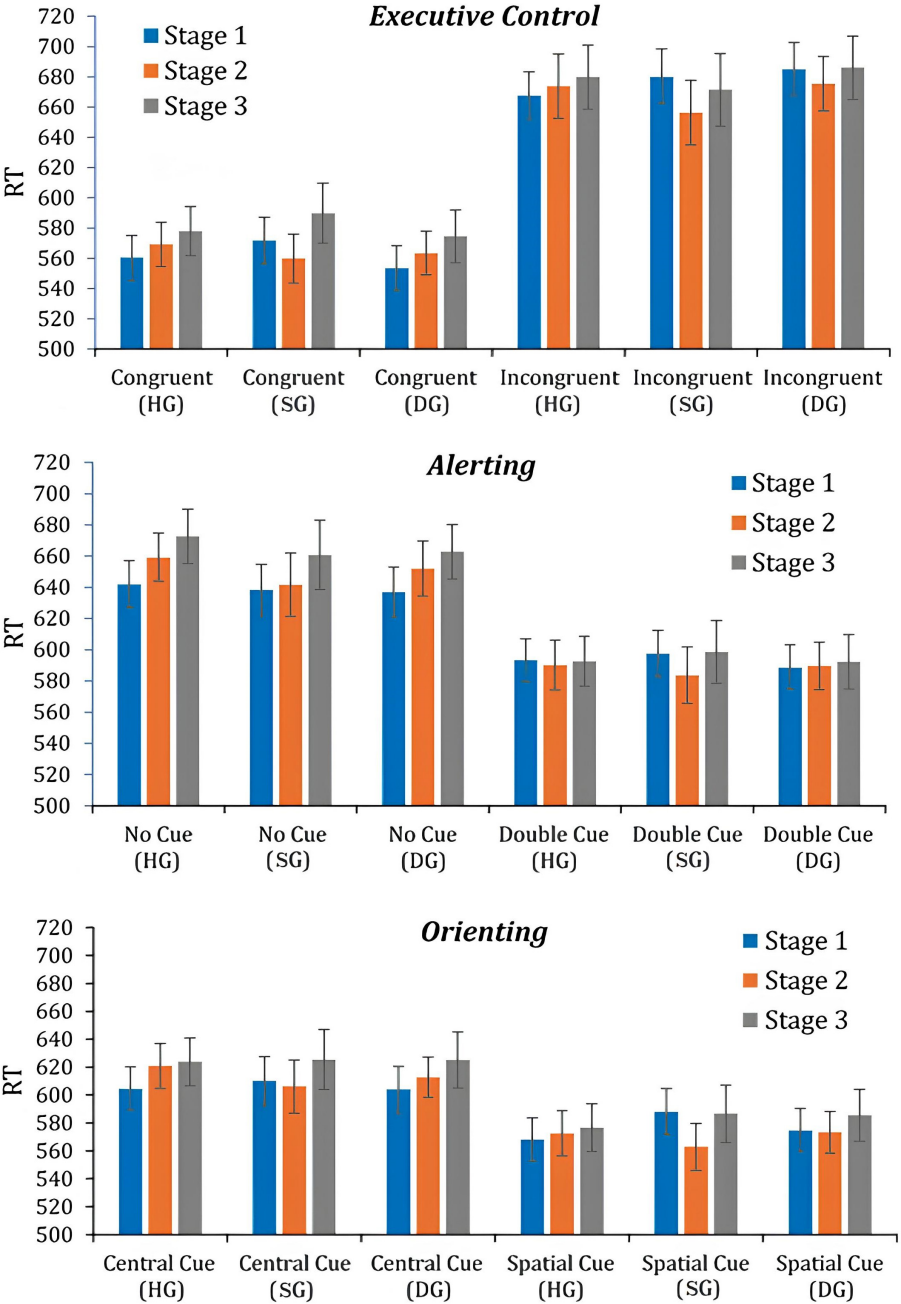
Mean RTs at three stages, in three groups (HG, SG, DG). RTs are shown for flankers/cues used in analyses of executive control (congruent and incongruent flankers), alerting (no cue and double cue), and orienting (central cue and spatial cue).

Attention Network Test performance data are shown in Figure 1; cell means may be found in Supplementary Appendix 1. Repeated-measures ANOVAs with a 2 × 3 × 3 (flanker/cue × group × stage) mixed-model design were run for each network. There were significant cue/flanker main effects for all three analyses. Response times were faster for: congruent versus incongruent flankers: (*F*(1,90) = 461.99, *p* < 0.01, *p*_*BH*_ < 0.01, η_*p*_^2^ = 0.849), double versus no cue (*F*(1,90) = 506.14, *p* < 0.01, *p*_*BH*_ < 0.01, η_*p*_^2^ = 0.837), and spatial versus center cue (*F*(1,90) = 274.74, *p* < 0.01, *p*_*BH*_ < 0.01, η_*p*_^2^ = 0.753). Overall means for the Fan et al. (2002) indices were 106 ms (Executive Control), 60 ms (Alerting), and 38 ms (Orienting). Supplementary Appendix 1 provides index values in each condition. The mean values suggest substantial flanker or cue effects for each network consistent with previous findings. There were no significant main or interactive effects of group in any of the analyses.

Near-significant though weak effects of stage were found in the analyses of flanker type (*F*(2,180) = 2.99, *p* = 0.053, *p*_*BH*_ > 0.10, η_*p*_^2^ = 0.032), double cueing (*F*(2,180) = 3.04, *p* = 0.050, *p*_*BH*_ > 0.10, η_*p*_^2^ = 0.033), and spatial cueing (*F*(2,180) = 3.03, *p* = 0.051, *p*_*BH*_ > 0.10, η_*p*_^2^ = 0.033). The flanker/cue × stage interaction was significant for flanker type (*F*(2,180) = 9.28, *p* < 0.01, *p*_*BH*_ < 0.01, η_*p*_^2^ = 0.093), double cueing (*F*(2,180) = 18.064, *p* < 0.01, *p*_*BH*_ < 0.01, η_*p*_^2^ = 0.167), and spatial cueing (*F*(2,180) = 8.47, *p* < 0.01, *p*_*BH*_ < 0.01, η_*p*_^2^ = 0.086). In each analysis, mean RT increased across stages in the respective control conditions (congruent flanker, no cue, center cue) but changed only slightly in the experimental conditions associated with the three networks (incongruent flanker, double cue, spatial cue). For example, in the executive control analysis, mean RT with congruent flankers increased from 562 to 581 ms (ΔRT = 19 ms) from stage 1 to stage 3 (data from whole sample). With incongruent flankers, the change was from 678 to 679 ms (ΔRT = 1 ms). This pattern of change produced temporal changes in the network indices (see Supplementary Appendix 1). It appears that over time there were improvements in executive control (index value decreases), alerting (index increases) and orienting (index increases). However, these index changes are misleading because they are driven by the slowing of RT in control conditions, and, consequently, a shift in the baseline used for the calculation of the index value.

### 3.3 ERP amplitude data

Event-Related Potential parameters included N100 amplitudes for responses to target and cue stimuli and P300 amplitudes for targets only. Data for executive control, alerting, and orienting networks were analyzed separately, using mean amplitudes from combined electrode sets as defined previously in the Section “2 Materials and methods.” Amplitudes were computed for each of the three task stages. We also analyzed frontal (Fz) response in a further set of analyses. The executive control data were analyzed using a 2 × 2 × 3 (flanker type × group × stage) mixed-model design, and alerting and orienting data using a 2 × 2 × 3 (cue type × group × stage) design. This section presents the ANOVA results as graphs, together with illustrative ERP plots and 2D topographic maps. The graphs average data across the three stages, for clarity. Key effects of stage are described in the text. Full tables of cell means may be found in Supplementary Appendix 2 (averaged across stages) and Supplementary Appendix 3 (broken out by stages). These tables also include the difference score indices for the three networks for descriptive purposes. These indices were not analyzed as outcome measures because difference scores can be misleading when there are temporal changes in baseline, which are common in tasks requiring sustained attention (Kustubayeva A. et al., 2022).

#### 3.3.1 Executive control network: ERP amplitudes

##### 3.3.1.1 P300 amplitude for congruent and incongruent flankers

**FIGURE 2:**
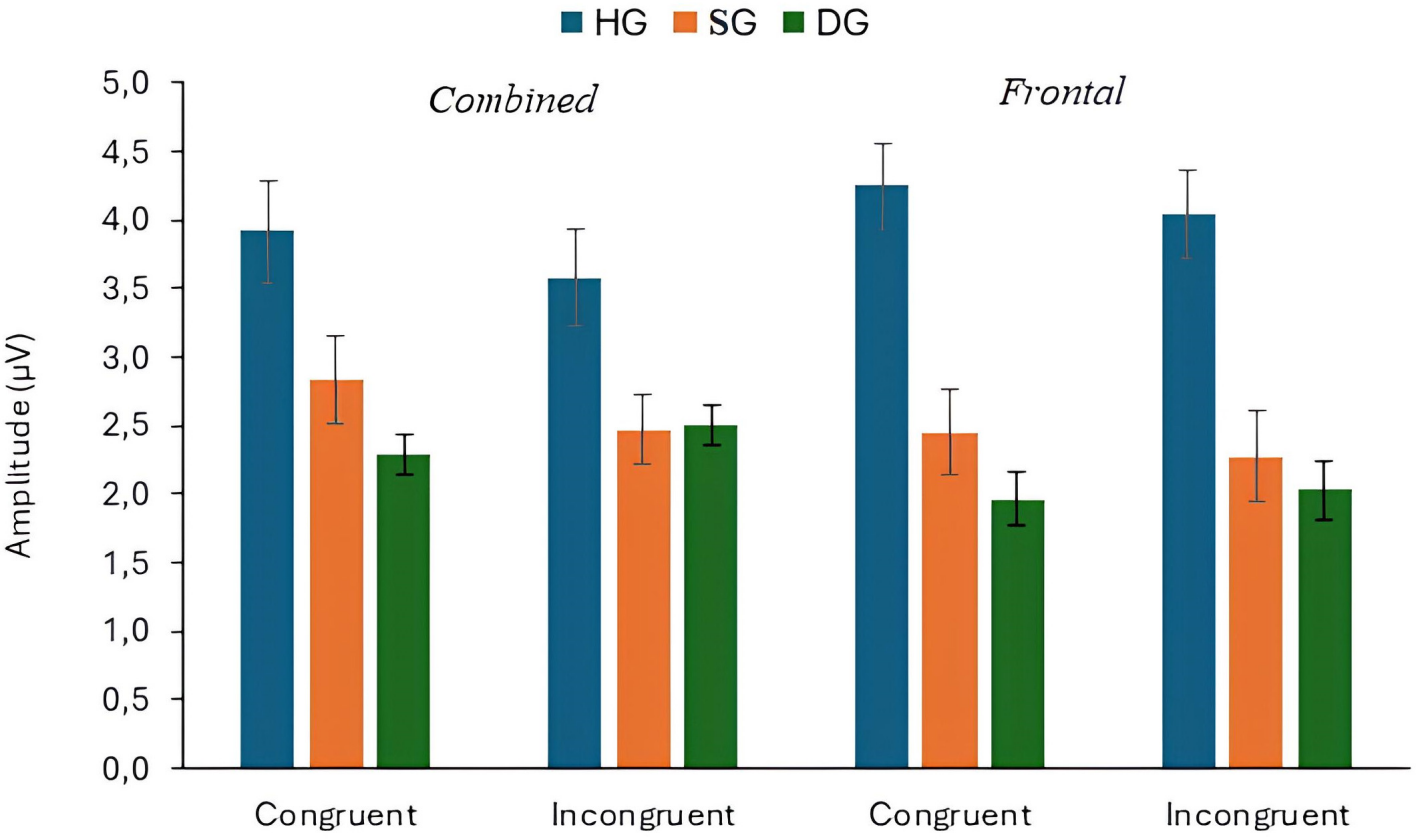
P300 response to target stimuli with congruent and incongruent flankers at combined and frontal sites, in three groups (HG, SG, DG).

**FIGURE 3:**
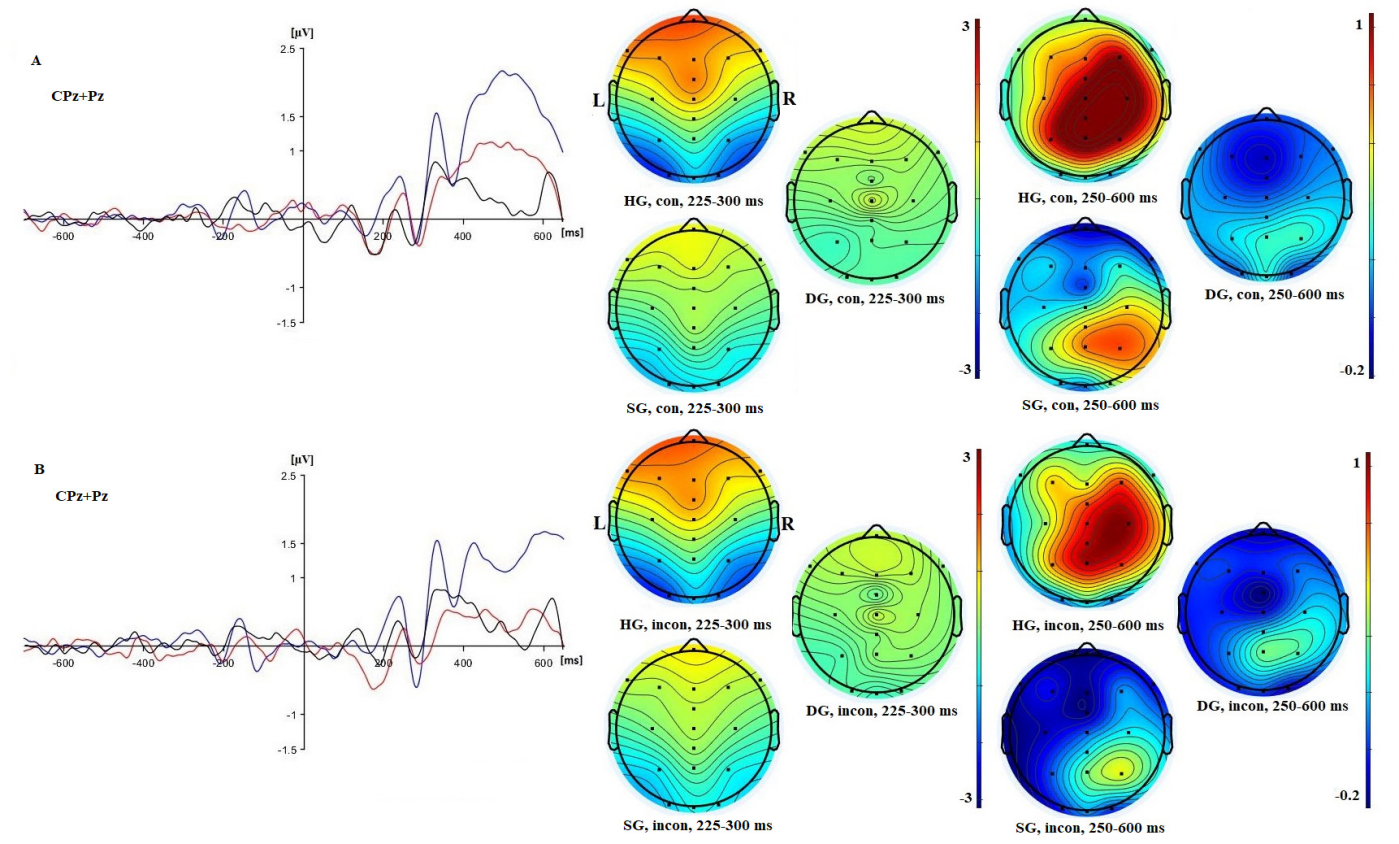
Left: ERPs for panel **(A)** congruent and panel **(B)** incongruent flanker conditions in three groups [in blue (HG), red (SG), and black (DG)] in combined electrodes (CPz + Pz)/2. Right: 2D maps of mean amplitude for congruent and incongruent flankers at time intervals representing N100 and P300 waves.

The mean amplitude of P300 responses to congruent and incongruent targets for the three groups are shown in Figure 2. The combined electrode data (mean amplitude at CPz and Pz) and Fz data are plotted separately. For the combined electrodes analysis of P300 target amplitude, there were significant main effects of flanker type (congruent/incongruent) (*F*(1,90) = 3.943, *p* = 0.050, *p*_*BH*_ < 0.10, η_*p*_^2^ = 0.042), group (*F*(2,90) = 7.006, *p* = 0.01, *p*_*BH*_ < 0.01, η_*p*_^2^ = 0.135), and stage (*F*(2,180) = 3.057, *p* < 0.05, *p*_*BH*_ < 0.10, η_*p*_^2^ = 0.033). As expected, P300 amplitudes were generally higher for congruent than for incongruent flankers, and the amplitude tended to decrease over time, although these effects were small in magnitude. The P300 response was larger for HG than for SG and DG. *Post hoc* Bonferroni tests analyzed using the SPSS GLM procedure showed that amplitude was significantly higher in HG than in SG, and higher in SG than in DG (*p* < 0.05). There were also significant flanker × group (*F*(2,90) = 4.847, *p* = 0.01, *p*_*BH*_ < 0.05, η_*p*_^2^ = 0.097), and stage × group (*F*(4,180) = 3.033, *p* < 0.05, *p*_*BH*_ < 0.05, η_*p*_^2^ = 0.063) interactions. The flanker × group interaction resulted from the flanker effect occurring in HG and SG but not in DG. Executive Control index values were 0.34 (HG), 0.36 (SG), and −0.20 (DG) (see Supplementary Appendix 2). Figure 3 illustrates the stronger P300 in HG in the voltage plots and topographic maps for the two flanker conditions. In fact, the effect is apparent across most of the scalp, not just at centro-parietal sites.

The main effect of stage reflected a tendency for combined-electrodes P300 amplitude to decline over time (see Supplementary Appendix 3). The stage × group interaction was associated with greater temporal decline in HG and DG than in SG. Averaging across flanker conditions, amplitude declined from 3.83 μV at stage 1 to 3.60 μV at stage 3 (Δ = −0.23) in HG. Temporal changes in the other groups were from 2.70 to 2.56 μV (Δ = −0.16) in SG, and from 2.71 to 2.44 μV (Δ = −0.27) in DG.

**FIGURE 4:**
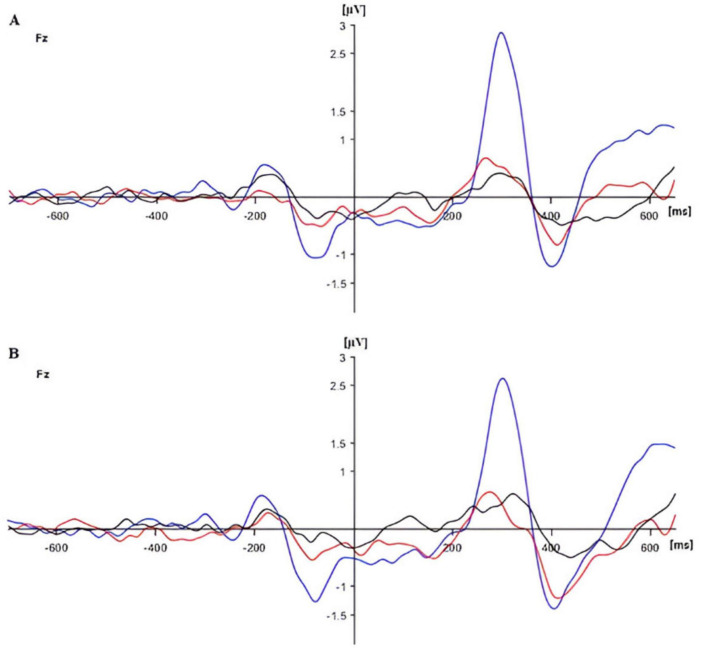
Event-Related Potentials (ERPs) for panel **(A)** congruent and panel **(B)** incongruent flanker conditions in three groups [in blue (HG), in red (SG), and in black (DG)] at Fz.

The additional analysis for the Fz electrode showed a significant group effect (*F*(2,90) = 17.576, *p* < 0.01, *p*_*BH*_ < 0.01, η_*p*_^2^ = 0.281). Frontal P300 amplitude was highest in HG and lowest in DG, as shown in Figures 2, 4. *Post-hoc* tests (*p* < 0.05) showed that HG differed from both SG and DG, and SG differed from DG. The group difference in frontal response can also be seen in the topographic maps of Figure 3. There were no other significant main or interactive effects in this analysis.

##### 3.3.1.2 Target N100 amplitude with congruent and incongruent flankers

**FIGURE 5:**
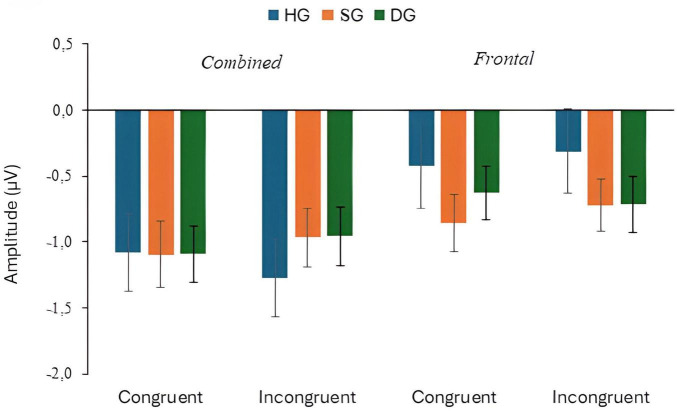
N100 response to target stimuli with congruent and incongruent flankers at combined and frontal sites, in three groups (HG, SG, DG).

Figure 5 shows N100 amplitude data for combined and frontal electrodes. The analysis for combined electrodes N100 amplitude 2 × 3 × 3 (flanker type × groups × stage) showed a significant stage effect (*F*(2,180) = 6.594, *p* < 0.01, *p*_*BH*_ < 0.05, η_*p*_^2^ = 0.068) but no significant flanker or group effects. N100 amplitude declined from −1.37 μV at stage 1 to 0.94 μV at stage 2 and 0.92 μV at stage 3. There were no significant effects of the experimental factors on response at Fz.

#### 3.3.2 Alerting network: ERP amplitude

##### 3.3.2.1 Cue and target N100 amplitudes in double cue and no cue conditions

**FIGURE 6:**
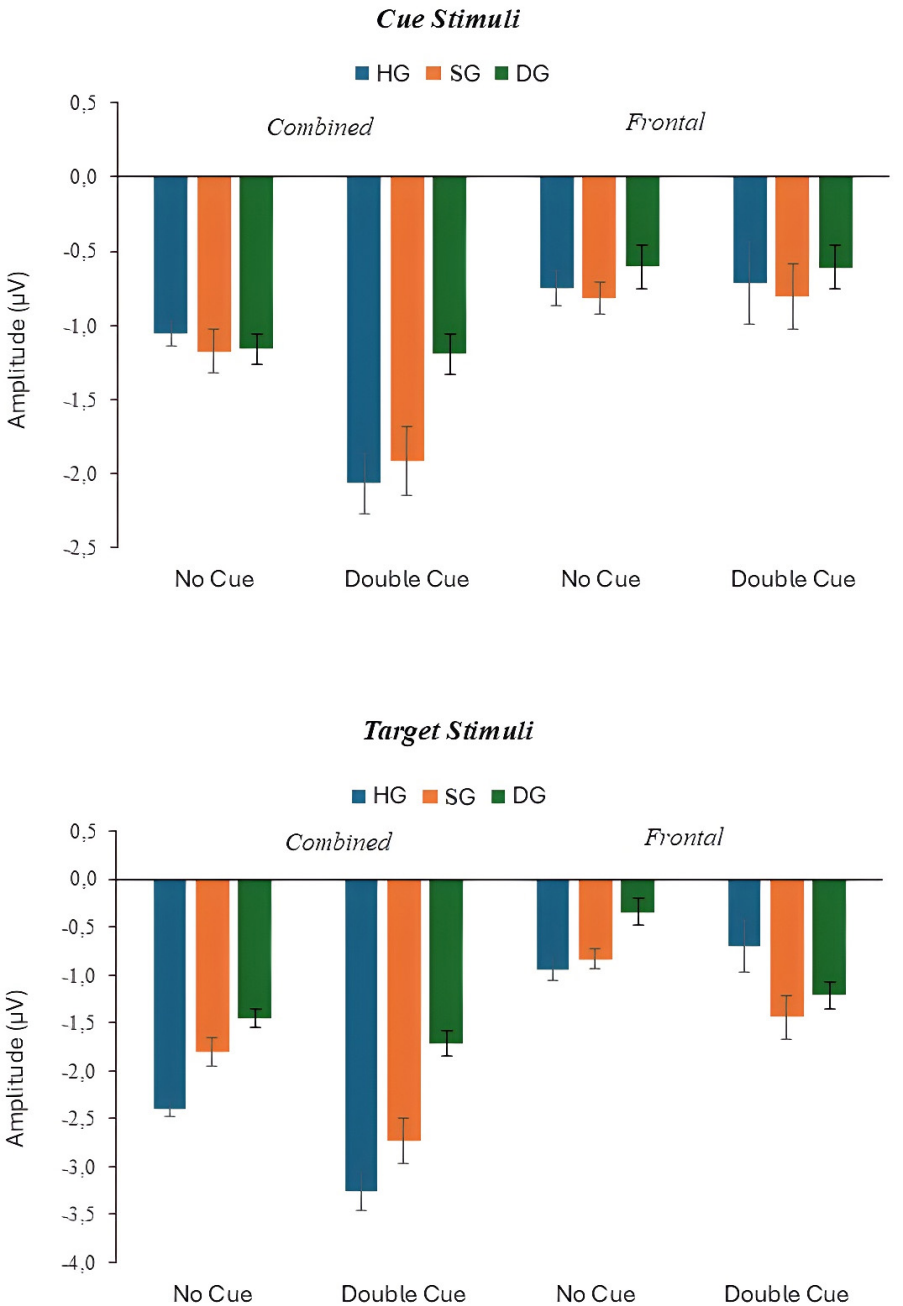
N100 response to cue and target stimuli with no cue and double cue at combined and frontal sites, in three groups (HG, SG, DG).

Alerting network response was indexed by averaging ERP amplitudes for combined electrodes Pz, P3, P4, O1, and O2. The means for N100 amplitude to cue and target, and P300 amplitude to target in three groups are shown in Figure 6. For N100 response to the cue, there were significant main effects of cue (*F*(1,90) = 22.896, *p* < 0.01, *p*_*BH*_ < 0.05, η_*p*_^2^ = 0.203), and group (*F*(2,90) = 3.419, *p* < 0.05, *p*_*BH*_ < 0.10, η_*p*_^2^ = 0.071), as well as a cue × group interaction (*F*(2,90) = 5.452, *p* < 0.01, *p*_*BH*_ < 0.01, η_*p*_^2^ = 0.108). The double cue elicited an N100 response, but only in HG and SG. Values of the alerting index were −1.01 (HG), −0.74 (SG), and −0.04 (DG) (see Supplementary Appendix 2).

**FIGURE 7:**
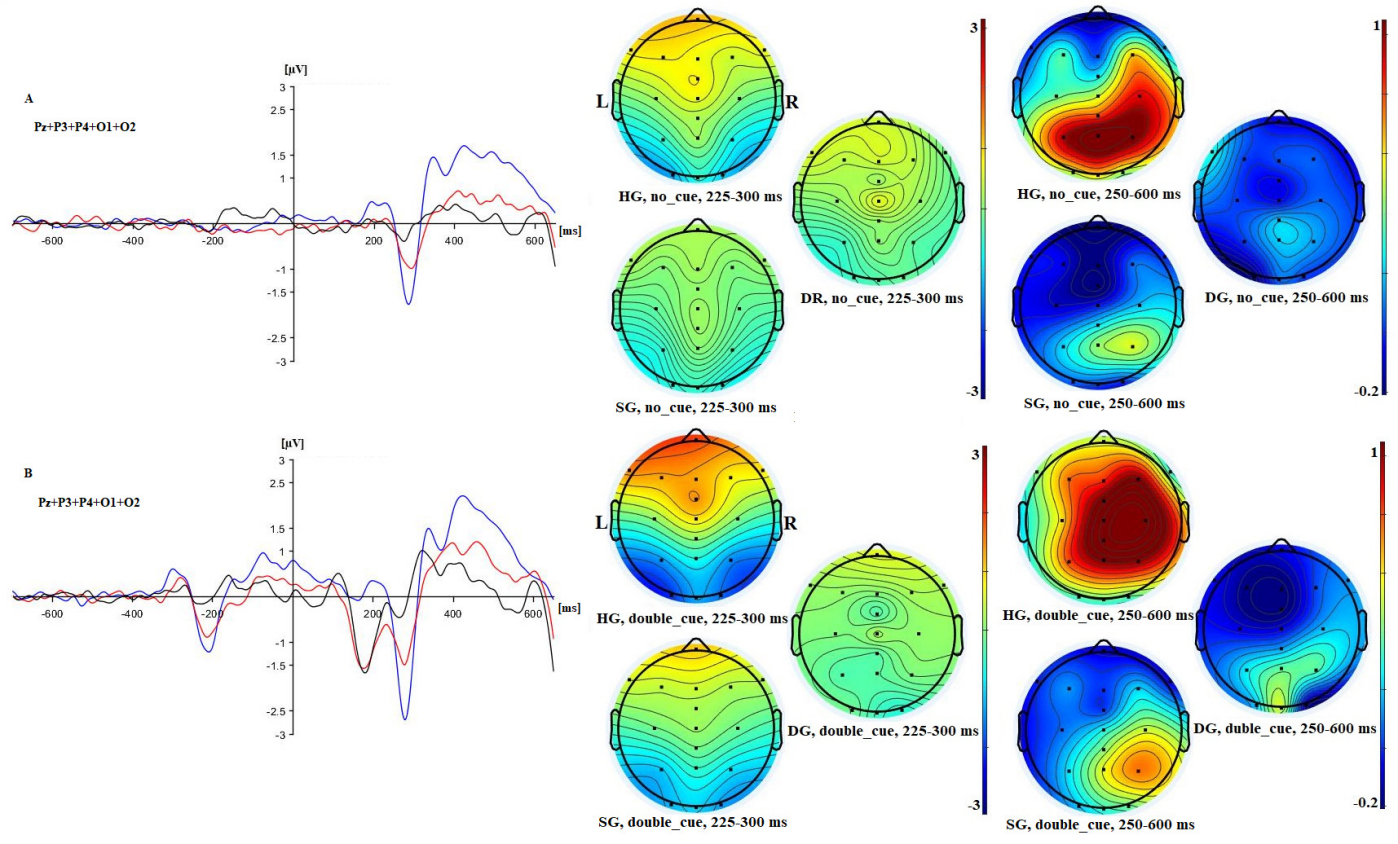
Left: ERPs for no cue **(A)** and double cue **(B)** conditions in three groups [in blue (HG), in red (SG), and in black (DG)] in combined electrodes (Pz, P3, P4, O1, O2)/5. Right: 2D maps of mean amplitude for congruent and incongruent flankers in three groups at time intervals representing N100 and P300 waves.

For target N100 there were significant main effects of cue (*F*(1,90) = 20.009, *p* < 0.01, *p*_*BH*_ < 0.01, η_*p*_^2^ = 0.182), group (*F*(2,90) = 4.236, *p* < 0.05, *p*_*BH*_ < 0.05, η_*p*_^2^ = 0.086), and stage (*F*(2,180) = 4.949, *p* < 0.01, *p*_*BH*_ < 0.05, η_*p*_^2^ = 0.052). The double cue elicited an N100 as expected. Averaging across cue conditions, potentials were most negative for HG, least negative for DG, and intermediate for SG. *Post-hoc* tests showed that all groups differed significantly (*p* < 0.05). There was a trend toward a stronger cuing effect in the HG and SG groups than for the DG group, but the cue × group interaction was not significant. The stage effect reflected a general reduction in negativity over time. Figure 7 further illustrates the larger HG response to both the initial cue and to the target.

The analyses of frontal (Fz) N100 showed generally weaker effects of experimental variables than the combined-electrodes analyses. For response to the cue, the only significant effect was the cue × stage interaction (*F*(2,180) = 4.457, *p* < 0.05, *p*_*BH*_ < 0.10, η_*p*_^2^ = 0.047). The cue produced a reduction in negativity at the third stage. For response to the target, there was a weak main effect of cue (*F*(1,90) = 4.627, *p* < 0.05, *p*_*BH*_ > 0.10, η_*p*_^2^ = 0.049), with the double cue producing a stronger negative wave. The main effect was moderated by a significant cue × group × stage interaction (*F*(4,180) = 3.960, *p* < 0.01, *p*_*BH*_ < 0.05, η_*p*_^2^ = 0.081). Temporal change in response to the cue varied across groups, but the pattern of change had no clear interpretation (see Supplementary Appendix 3 for cell means).

##### 3.3.2.2 Target P300 amplitude in double cue and no cue conditions

**FIGURE 8:**
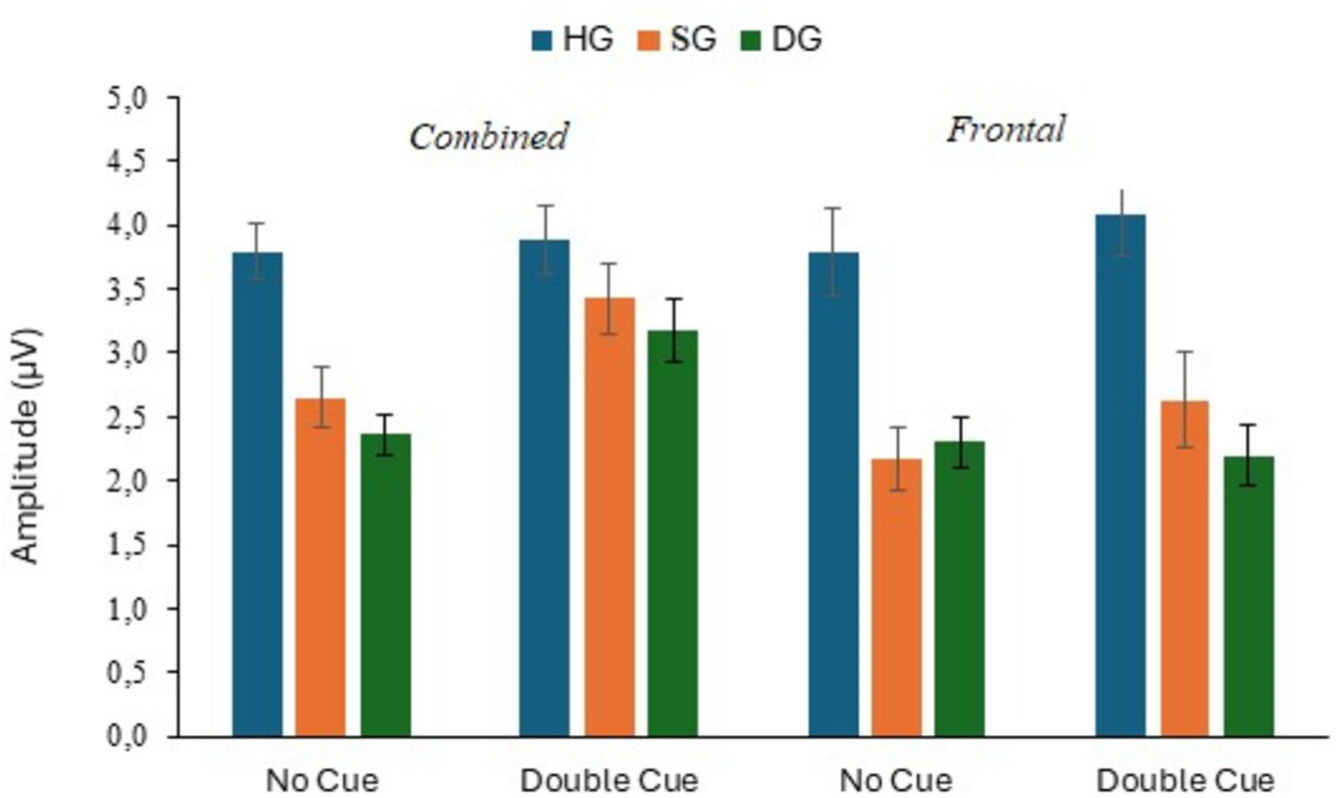
P300 response to target stimuli with no cue and double cue at combined and frontal sites, in three groups (HG, SG, DG).

Analysis of P300 amplitude for the combined electrodes showed a significant cue effect (*F*(1,90) = 15.961, *p* < 0.01, *p*_*BH*_ < 0.01, η_*p*_^2^ = 0.151) associated with a positive response to the cue (see Figure 8). The group effect was significant (*F*(2,90) = 7.543, *p* < 0.01, *p*_*BH*_ < 0.01, η_*p*_^2^ = 0.144). Amplitude values were highest in HG and lowest in DG, with intermediate values in SG, in both uncued and cued conditions. There was also a significant main effect of stage (*F*(2,180) = 6.935, *p* < 0.01, *p*_*BH*_ < 0.01, η_*p*_^2^ = 0.072); voltages tended to decline across both conditions and in all three groups. There were no significant interactions between factors.

**FIGURE 9:**
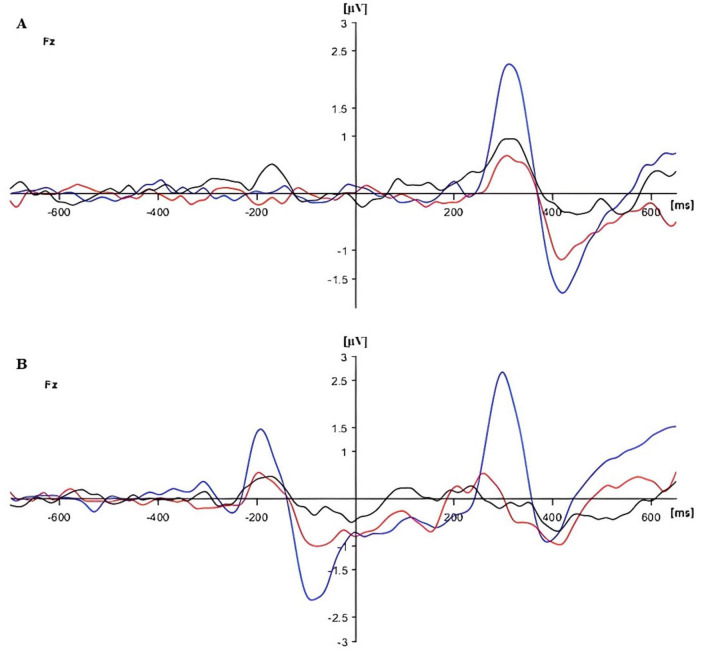
Event-Related Potentials (ERPs) for no cue **(A)** and double cue **(B)** conditions in three groups [in blue (HG), in red (SG), and in black (DG)] at Fz.

The analysis of Fz response showed only a main effect of group (*F*(2,90) = 12.035, *p* < 0.01, *p*_*BH*_ < 0.01, η_*p*_^2^ = 0.211). The P300 response was considerably stronger in the HG group than in the other two groups, as illustrated in Figure 9.

#### 3.3.3 Orienting network: ERP amplitude

##### 3.3.3.1 Cue and target N100 amplitude in spatial cue and central cue conditions

**FIGURE 10:**
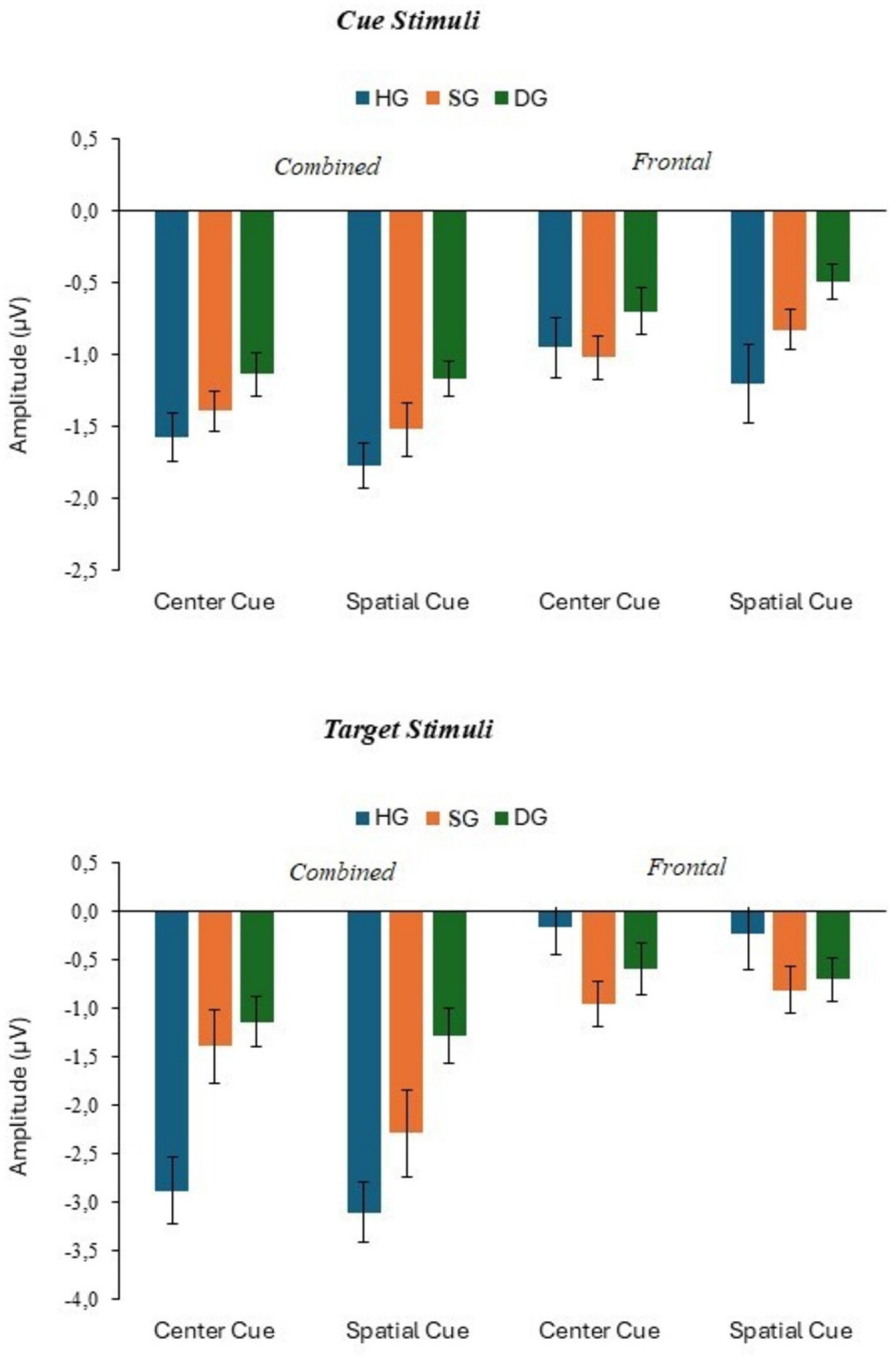
N100 response to cue and target stimuli with center cue and spatial cue at combined and frontal sites, in three groups (HG, SG, DG).

The analyses of the orienting network were based on the combined Pz, P3, P4, O1, and O2 electrode response and the spatial cueing manipulation (see Figure 10). For N100 response to the cue, the 2 × 3 × 3 (spatial cue/central cue × group × stage) ANOVA showed significant but weak main effects of group (*F*(1,90) = 3.712, *p* < 0.05, *p*_*BH*_ > 0.10, η_*p*_^2^ = 0.076) and stage (*F*(2,180) = 3.127, *p* < 0.05, *p*_*BH*_ > 0.10, η_*p*_^2^ = 0.034). The spatial cueing manipulation had minimal effects on N100 response. HG had the strongest responses to both central and spatial cues and DG the weakest, as shown in Figure 5. Amplitude tended to decrease over time, especially from stage 1 to stage 2 (see Supplementary Appendix 3).

**FIGURE 11:**
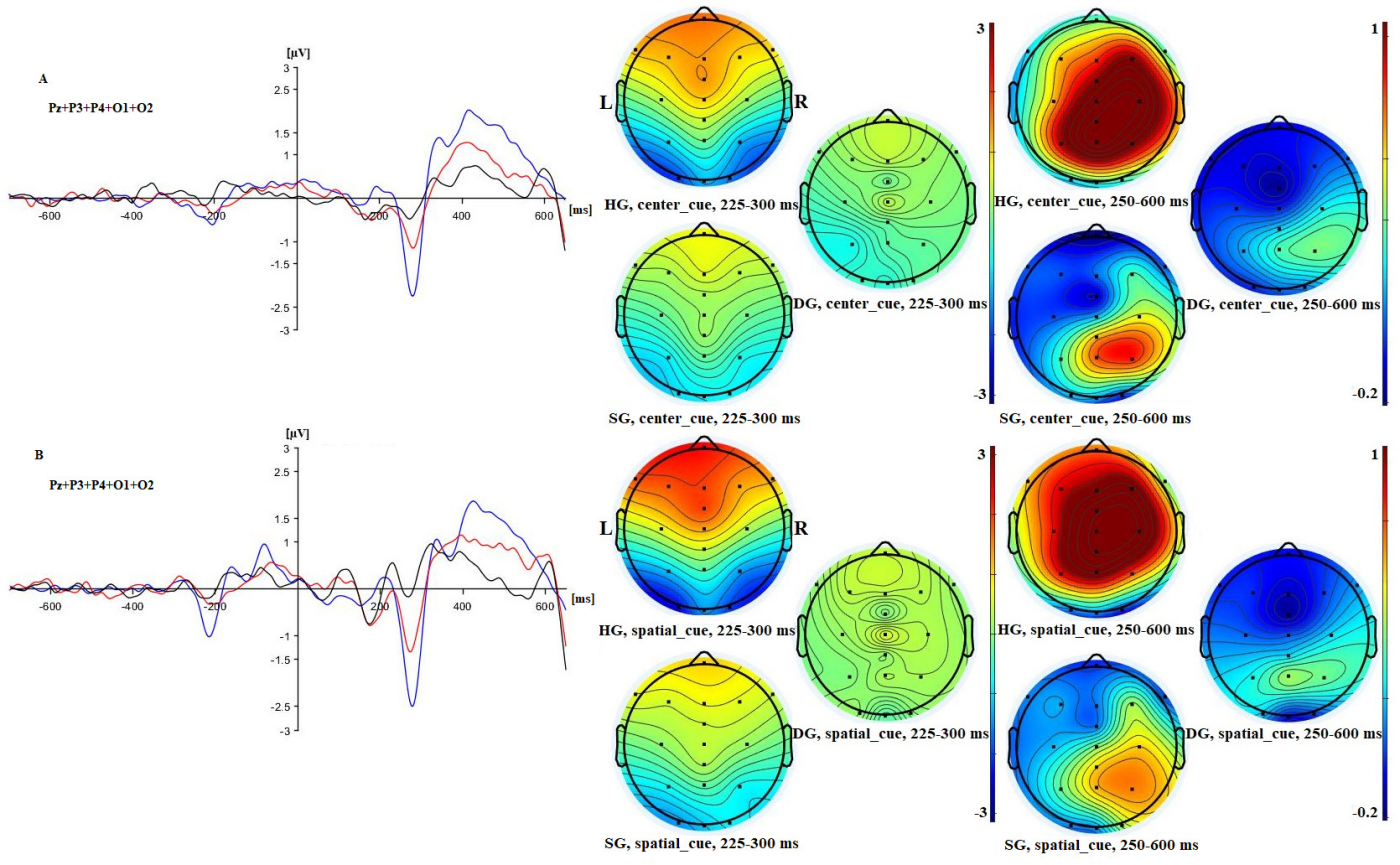
Left: ERPs for center cue **(A)** and spatial cue **(B)** conditions in three groups [in blue (HG), in red (SG), and in black (DG)] in combined electrodes (Pz, P3, P4, O1, O2)/5. Right: 2D maps of mean amplitude with center and spatial cues in three groups at time intervals representing N100 and P300 waves.

The analysis of response to the target showed significant main effects of group (*F*(2,90) = 5.644, *p* < 0.01, *p*_*BH*_ < 0.05, η_*p*_^2^ = 0.111) and stage (*F*(2,180) = 7.969, *p* < 0.01, *p*_*BH*_ < 0.01, η_*p*_^2^ = 0.081) and a stage × group interaction (*F*(4,180) = 3.067, *p* < 0.05, *p*_*BH*_ < 0.05, η_*p*_^2^ = = 0.064). As for response to the cue, amplitude was highest for HG and lowest for DG, irrespective of cue type (see Figure 11). Temporal change differed across groups. For HG, amplitude decreased from 3.58 μV at stage 1 to 2.71 μV at stage 3 (Δ = −0.87) and for SG from 2.61 to 2.18 μV (Δ = −0.43). Amplitude changed little from 1.38 μV at stage 1 to 1.37 μV at stage 3 (Δ = −0.01) in DG. That is, the temporal amplitude decline was strongest in HG but amplitude was still greater for HG than for DG at stage 3.

**FIGURE 12:**
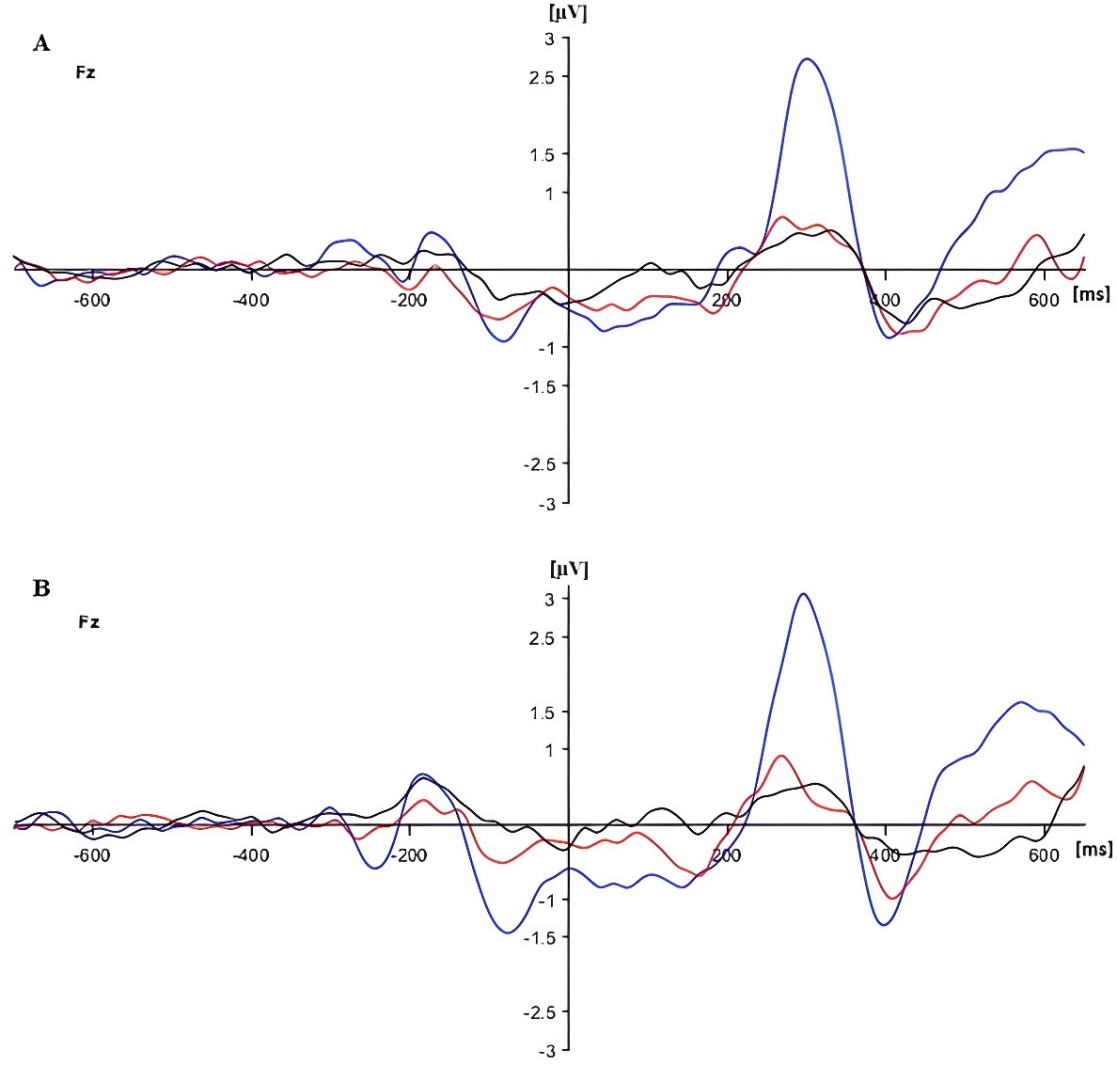
Event-Related Potentials (ERPs) for center cue **(A)** and spatial cue **(B)** conditions in three groups [in blue (HG), in red (SG), and in black (DG)] at Fz electrode.

There were no significant effects of the experimental factors on frontal N100 response, which likely reflects the absence of the wave at Fz (see Figure 12).

##### 3.3.3.2 P300 amplitude in spatial cue and central cue conditions

**FIGURE 13:**
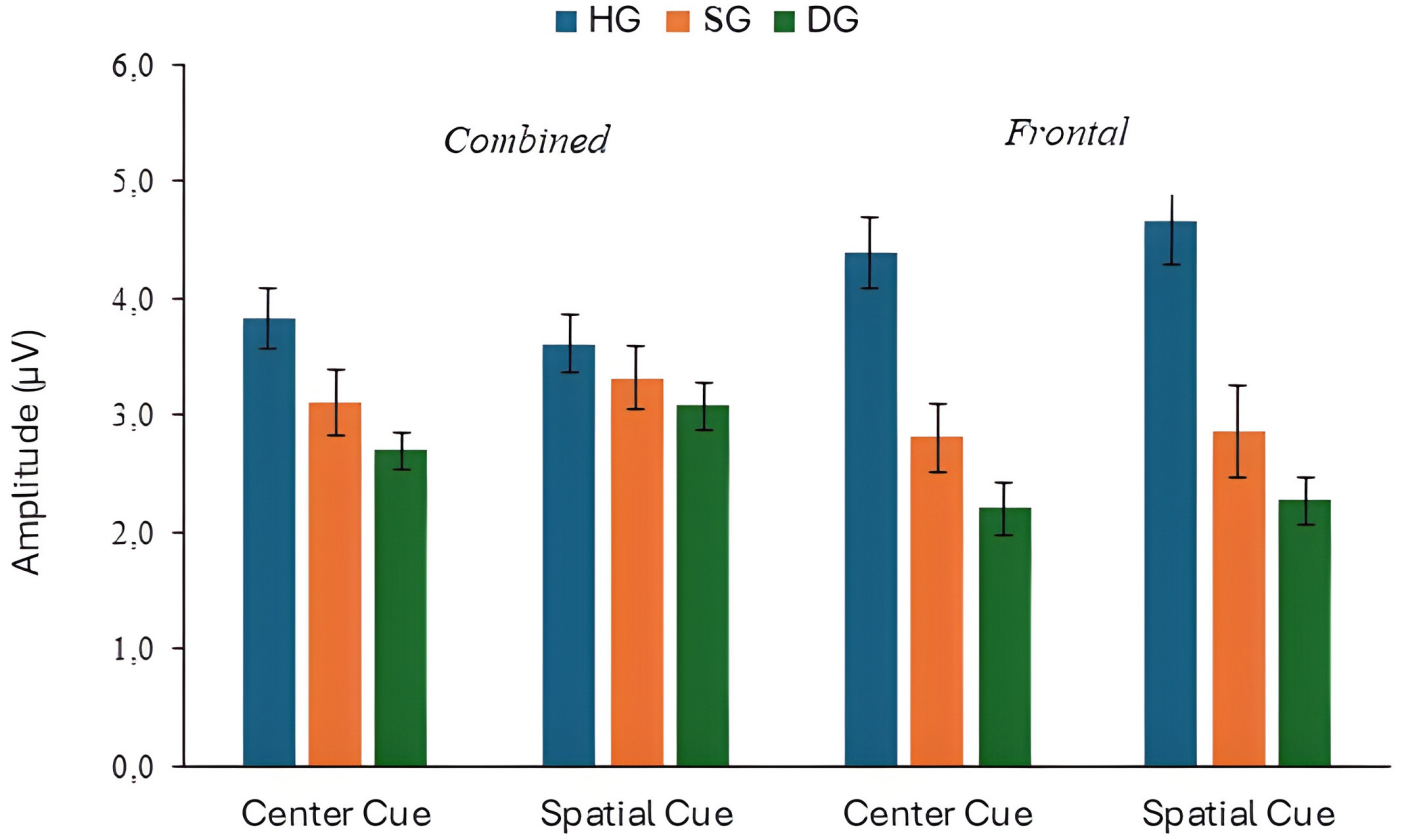
P300 response to target stimuli with center cue and spatial cue at combined and frontal sites, in three groups (HG, SG, DG).

The analysis for the combined electrodes revealed a significant group effect (*F*(2,90) = 3.514, *p* < 0.05, *p*_*BH*_ < 0.10, η_*p*_^2^ = 0.072), a stage effect (*F*(2,180) = 3.762, *p* < 0.05, *p*_*BH*_ < 0.10, η_*p*_^2^ = 0.040), and a cue × group interaction (*F*(2,90) = 3.778, *p* < 0.05, *p*_*BH*_ < 0.10, η_*p*_^2^ = 0.077), although these effects were all small in magnitude. The group effect reflected HG showing the highest P300 response in both cue conditions, which can be seen in Figures 11, 13. The interaction was due to a somewhat larger group effect in the center cue relative to the spatial cue condition. However, Orienting index values were generally close to zero showing that the spatial cue did not markedly influence the P300. The main effect of stage was associated with temporal decline in amplitude: means were 3.37, 3.36 and 3.09 μV for stages 1, 2 and 3.

The only significant effect in the analysis of Fz response was a main effect of group (*F*(2,90) = 16.258, *p* < 0.01, *p*_*BH*_ < 0.01, η_*p*_^2^ = 0.265). P300 amplitude was highest in HG and lowest in DG, as shown in Figure 6.

## 4 Discussion

The present study examined effects of depression and temporal stage on behavioral and ERP responses to a long-duration version of the ANT. Behavioral data including cue and flanker effects on response times were consistent with Fan et al.’s (2002) findings. That is, alerting and orienting cues speeded response and incongruent flankers slowed response. Effects of task factors on ERP amplitude were also consistent with previous studies (Kustubayeva A. et al., 2022; Neuhaus et al., 2010). These included reduced central-parietal P300 response to incongruent flankers, increased parietal-occipital response to the double cue, and no clear impact of the spatial cue. We also found evidence for loss of sustained attention over time, including slowing of response in control conditions, and decreasing N100 and P300 amplitude in multiple analyses, similar to Kustubayeva A. et al. (2022).

The principal hypothesis was that depressed patients would show ANT executive control impairments. We confirmed that DG experienced impaired mood during task performance but there was no significant impact of depression on the behavioral data. There were pervasive effects of depression on ERP amplitude, especially over the electrode sites identified with the Petersen and Posner (2012) networks. Frontal P300 amplitude was also lower in DG. We found an interactive effect of depression and flanker type on central-parietal P300, consistent with our hypothesis. However, the form of the interaction was different to expectation, as further discussed below. We also found reduced N100 response to the double cue in DG, suggesting impaired alerting in depression. We did not find any meaningful interaction between depression and temporal declines in ERP amplitude.

### 4.1 Depression, ERPs and attention

There were substantial differences between MDD patients and healthy controls on multiple ERP amplitude measures. Previous authors (e.g., Greco et al., 2021) have commented that depression effects on ERPs may be task-specific, contributing to inconsistency across studies. The ANT (Fan et al., 2002) appears to be successful in eliciting depression effects on both earlier and later ERPs. Depression effects were more widespread for analyses based on the scalp electrode sets associated with the three Petersen and Posner (2012) networks than for frontal analyses, although depression influenced frontal P300. In the executive control analysis (combined electrodes), the effect sizes (Cohen’s *d*) for comparison of P300 means between HG and DG were 1.07 (congruent flankers) and 0.71 (incongruent flankers). For the N100 response to target, effect sizes were 0.64 (no cue) and 0.83 (double cue), i.e., medium to large effect sizes. They appear to exceed the medium effect size (0.49) found in Arıkan et al.’s (2024) meta-analysis of depression effects on the oddball task. They suggest a pervasive attentional deficit in depression consistent with previous behavioral (Ahern and Semkovska, 2017), neurological (Keller et al., 2019) and fMRI (Pilmeyer et al., 2022) studies.

Depression effects on the Fan et al. (2002) network indices were smaller in magnitude. There was a significant interaction between depression group and flanker type for P300 amplitude. Paradoxically, the Executive Control index values suggested stronger control in DG than the other groups (Figure 2), but in this instance the index may be misleading. The depressed individuals showed a strongly attenuated P300 with congruent flankers which is likely to have limited further reduction in amplitude with incongruent flankers. It is also possible that the deficit in inhibitory attention associated with depression (Kircanski et al., 2012) resulted in the congruent as well as the incongruent flankers being distracting for DG.

The effect of depression on alerting was more straightforward: healthy controls showed a substantially enhanced N100 response to the double cue, but DG showed only a small response. This effect was apparent both for immediate response to the cue and for response to the subsequent target stimulus. Depressed individuals appear to be deficient in selective attention to the cue and target as well as to the later cognitive processing indexed by P300.

It is perhaps surprising that depression had no impact on the behavioral data given the robust ERP findings. ERPs may be more sensitive to neurocognitive deficits in depression than the RT-based measures are. The current findings also reflect the modest impact of depression on standard ANT indices. Sinha et al.’s (2022) review found a median difference of 19 ms between depressed and healthy individuals on the Executive Control index. In this study, the comparable data are for DG and HG in the first phase of the study, which is a similar task duration to the standard ANT. Values of the index (see Table 1) were 132 for DG and 107 for HG, a 25 ms difference which is within Sinha et al.’s (2022) confidence interval for the effect. Cohen’s *d* for the difference was 0.51. The non-significance in the ANOVA likely reflects high variability in the DG group and the attenuation of the difference across time to a 9 ms difference at stage three. Effects were insufficiently strong to produce either a flanker × depression interaction or a flanker × stage × depression interaction.

The study also included a “subsyndromal” group with elevated IDS scores but no psychiatric diagnosis. Broadly, an ERP abnormality shared by SG and DG might indicate an underlying neurocognitive risk factor, while one seen in DG only might be a concomitant of the depressed state. In general, SG and DG showed qualitatively similar patterns of ERP response, especially for P300, but some differences between the two groups signal which ERP metrics are most promising as biomarkers for vulnerability to MDD. Specifically, P300 response in SG was intermediate in amplitude compared to the other two groups. *Post-hoc* tests showed that amplitude for SG was significantly different from both HG and DG at both parieto-central and frontal sites (see Figure 2). Thus, reduced P300 amplitude in individuals with depressive symptoms who do not meet the criteria for a major depressive episode might indicate a vulnerability to clinical depression. However, Executive Control index values were similar in HG and SG, suggesting any specific network deficit might be a marker of current depression. Effects of depression on N100 differed for target and cue response. The response to the target was smaller in DG and SG than in HG but SG and DG did not differ, according to the *post-hoc* tests (see Figure 6). Thus, like P300, overall N100 response amplitude may be indicative of vulnerability to depression. By contrast, HG and SG showed similar N100 alerting effects to the double cue, in both the cue and target analyses, whereas DG participants were unresponsive to the cue. Thus, the alerting effect may not provide a biomarker for vulnerability to depression. Instead, insensitivity to the alerting cue may be associated with the wider range of symptoms associated with MDD compared with subsyndromal depression, suggesting a state rather than a trait effect. Further investigation would be necessary to determine whether any specific symptoms or components of the state are associated with the N100 effect.

### 4.2 Temporal decline in attentional networks

Fatigue is a common clinical feature of depression, and it may mediate depression effects on executive function (Kraft et al., 2023). This study used (low) energetic arousal as an index of state fatigue. Depressed patients show substantially lower energetic arousal (Matthews and Southall, 1991), an effect confirmed in the current data. The task engagement state that includes high energetic arousal is positively associated with executive control on the ANT (Matthews and Zeidner, 2012), and with performance on a range of other demanding attentional tasks (Matthews, 2021). We expected that depression effects would become progressively stronger as participants became more fatigued during the extended performance interval.

Data showed loss of sustained attention over time. In the behavioral data, response slowed in control conditions, but not where there was an alerting or central cue, or flankers were incongruent. Kustubayeva A. et al. (2022) also found greater slowing of response in ANT control conditions. This result may suggest a general loss of attentional resources over time, consistent with other findings on vigilance (Warm et al., 2008), with no specific temporal deficit in the Petersen and Posner (2012) networks. Main effects of stage on N100 and P300 amplitude were found in all three analyses, although not all were significant with the Benjamini-Hochberg correction applied. Stage effects appeared to be most consistent across analyses of N100 response to the target. Amplitudes of both waves decreased over time, in multiple flanker/cue conditions. Temporal reduction in N100 and P300 amplitude has been observed in other sustained attention studies, reviewed by Arnau et al. (2021), and in our previous study (Kustubayeva A. et al., 2022).

However, despite the sensitivity of ERP measures to time on task, there was no differential effect on depressed individuals. Where stage and group interacted, effects were of small magnitude and hard to interpret. For example, the stage × group interaction for P300 in the executive control analysis was associated with a larger amplitude decline in SG compared to both HG and DG, an effect that has no obvious interpretation. It appears that depression and time on task effects are largely additive, and depressed individuals have no specific vulnerability to temporal impairments in attention network functioning. The lack of sensitivity of depression effects to time on task suggests effects might be more strongly related to trait than to state factors.

### 4.3 Clinical relevance

Researchers have identified three potential applications of P300 assessment in treatment of depression. First, identification of reliable biomarkers for depression can enhance diagnosis by complementing assessment of subjective symptoms with objective indices for brain dysfunction (Arıkan et al., 2024). EEG methods readily transition to clinical practice due to the portability, cost-effectiveness and scalability of modern systems (Etkin and Mathalon, 2024). A challenge is that abnormal ERPs are seen in a wide range of clinical disorders (Raggi et al., 2024) and research is necessary to establish biomarkers specific to depression and subtypes of depression. Second, P300 response may predict the patient’s response to therapy (Baskaran et al., 2012; Santopetro et al., 2020; Wade and Iosifescu, 2016). Research on this issue has been limited and the ERP predictors of treatment response found in existing studies are heterogeneous (Baskaran et al., 2012; Wade and Iosifescu, 2016). Santopetro et al. (2020) cite three studies linking pre-treatment P300 amplitude to better outcomes following treatments including antidepressants and electroconvulsive therapy (ECT). Given that behavioral measures of executive function predict poor response to antidepressants (Dunkin et al., 2000), ERP metrics linked to executive control such as P300 may be promising for future research. Third, P300 amplitude may track the course of depression in naturalistic settings (Santopetro et al., 2020). These authors measured P300 response to a flankers task similar to that use to assess executive control in the ANT. Lower P300 amplitude predicted severity of depression symptoms 9 months later. Santopetro et al. (2020) averaged response across congruent and incongruent flanker trials consistent with the present finding that P300 responses to each flanker type appear to be similarly diagnostic of depression. The present study was not designed to address treatment issues. However, findings underscore the potential importance of using validated attentional tasks such as the ANT to elicit the P300 in clinical studies.

### 4.4 Limitations

Several limitations should be noted. First, reviews of EEG biomarkers for depression have identified multiple types of biomarkers in addition to ERPs, including spectral power analysis of EEG frequency bands, lateralization of EEG response, source resolution using low-resolution electromagnetic tomography (LORETA), complexity metrics, functional connectivity, machine learning analysis of large data sets, and additional signal-based and network-based features (De Aguiar Neto and Rosa, 2019; Greco et al., 2021; Pilmeyer et al., 2022; Wade and Iosifescu, 2016). The plethora of metrics is currently challenging the capacity of researchers to test them against each other as correlates and predictors of depression. P300 amplitude appears promising as an EEG biomarker but there may be better ones. Second, ERP responses may vary across depression subtype (Bruun et al., 2021), but we did not have a sufficiently large sample to discriminate subtypes. Third, the present study addressed effects of depression on an attentional task utilizing affectively neutral stimuli. Research has long recognized both cognitive deficits and cognitive biases toward emotional stimulus content as features of depression (LeMoult and Gotlib, 2019; Wells and Matthews, 1994). Studies using emotive word or facial stimuli have found that depression effects can be moderated by emotional content (see Greco et al., 2021, for a review). Contrasting with the current findings, studies have reported that depression elevates P300 amplitude response to negative words (Ilardi et al., 2007) and to emotive stimuli on a face-word Stroop task (Zhu et al., 2018). Further work is needed to determine how ERP research can best be utilized to explore the relationship between deficits and bias (Ilardi et al., 2007).

Fourth, an attraction of using the ANT for depression research is the clear psychometric distinction between the indices for the three attentional networks (Fan et al., 2002), although there is some interaction between systems (Xuan et al., 2016). However, recent analyses of functional connectivity in brain networks have proposed that multiple, overlapping networks may support attentional control including an extended frontoparietal and cingulo-opercular networks (Dosenbach et al., 2008). The frontoparietal network, which contributes to a range of attentional and working memory functions, appears to contribute to both alerting and executive control (Markett et al., 2014). It is also implicated in a range of clinical conditions including depression (Cole et al., 2014). The wide-ranging effects of depression on N100 and P300 found in the present study might be characterized in relation to frontoparietal or other networks rather than the Petersen and Posner (2012) networks. However, identification and discrimination of networks remains challenging (Markett et al., 2022). In addition, the ANT does not assess components of executive function beyond response inhibition such as working memory updating and set shifting (Miyake et al., 2000), which merit further investigation. Other executive function measures might be more diagnostic of depression.

Fifth, we used scores on the IDS (Rush et al., 1996) to identify a subsyndromal depression group that reported high levels of depressive symptoms but did not attain full criteria for the diagnosis of MDD. However, subsyndromal (or subthreshold) depression has been defined and measured differently in different studies, leading to uncertainties over the nature of the construct (Rodríguez et al., 2012; Volz et al., 2023). Researchers have used both dimensional measures of depression, as here, and presence of a small number of symptoms of MDD (<5) to identify cases (Volz et al., 2023). Thus, use of the IDS to identify subsyndromal depression may be questionable. In defense of the current usage, Karsten et al. (2010) found that the IDS was more strongly correlated with functional impairment than was a symptom count in a subsyndromal sample. Furthermore, the high 2-years test-retest reliability of the IDS (Jeronimus et al., 2013) implies that it measures a stable tendency toward depression as well as current symptoms. The IDS is also substantially correlated with neuroticism (Spinhoven et al., 2011), a stable trait that is a known vulnerability factor for depression (Williams et al., 2021). However, other conditions such as grief, and chronic stress may cause individuals to report symptoms that overlap with those present during a major depressive episode that may not be directly linked to biological and psychological factors that produce a vulnerability for developing full MDD. Thus, further refinement of methods to identify subsyndromal depression is necessary to define biomarkers for vulnerability to future depression.

## 5 Conclusion

Patients with MDD showed substantial reductions in N100 and P300 response during performance of an extended-duration ANT. Depression was also associated with deficits in executive control and alerting as defined by Fan et al. (2002), but these network-specific effects were smaller in magnitude than the more generalized effects on amplitude also observed. Neurocognitive deficits in depression may relate to depletion of a general attentional resource or to impairments in an extended frontoparietal network supporting multiple attentional functions. Parieto-central and frontal P300 responses also tended to be attenuated in individuals with subsyndromal depression suggesting they may represent biomarkers for a vulnerability to developing major depressive disorder. ERP responses can potentially support clinical applications such as support for diagnosis and anticipation of treatment outcomes, but further research is necessary to elucidate relationships between functional brain networks, ERPs, and performance on the ANT.

## Significance

Findings contribute to the identification of clinically useful biomarkers for depression.

## Data Availability

The data analyzed in this study is subject to the following licenses/restrictions: restrictions by the Ethics Committee. Requests to access these datasets should be directed to AK, almkusto@kaznu.kz.
