# Supplementary material for: Event-Related Potentials and executive control deficits in major depression: evidence from the Attention Network Test
Source: Front Syst Neurosci. 2026 Jan 16;19:1632849. doi: 10.3389/fnsys.2025.1674124 (PMC12856934; doi:10.3389/fnsys.2025.1674124)
Supplement: Supplementary file 1 [file Table_1.docx]

**Appendix 1. ANT Reaction Time Data**

**Table A1.1.**

Means (and SDs) of reaction time (RT, ms) by flanker/cue type for each stage, group and overall. Index values were calculated as difference scores according to Fan et al. (2002).

|  | | Group | | | | | | | | | Overall |
| --- | --- | --- | --- | --- | --- | --- | --- | --- | --- | --- | --- |
|  |  | Healthy | | | Subsyndromal | | | MDD | | |  |
|  |  | 1st | 2st | 3st | 1st | 2st | 3st | 1st | 2st | 3st |  |
| Flanker | Congruent | 560.25 (82.52) | 569.25 (86.88) | 578.03 (82.53) | 571.74 (81.31) | 559.83 (89.63) | 589.79 (80.65) | 553.48 (90.23) | 563.32 (111.13) | 574.61 (96.80) | 568.93 (83.27) |
|  | Incongruent | 667.43 (87.87) | 673.70 (102.22) | 679.88 (97.83) | 680.11 (119.78) | 656.28 (118.82) | 671.46 (116.01) | 685.16 (118.06) | 675.47 (133.71) | 686.01 (116.01) | 675.06 (103.85) |
|  | Executive Control Index | 107.08 (37.80) | 104.45 (34.25) | 101.85 (36.31) | 108.38 (80.59) | 96.46 (58.75) | 81.67 (49.64) | 131.68 (57.23) | 112.15 (49.35) | 111.40 (55.34) | 106.12 (48.04) |
| Alerting Cue | No Cue | 641.68 (84.19) | 659.10 (92.09) | 672.56 (90.25) | 638.21 (86.55) | 641.71 (112.54) | 660.73 (98.25) | 636.75 (97.09) | 652.04 (123.08) | 662.80 (107.33) | 651.75 (91.90) |
|  | Double Cue | 593.39 (75.78) | 589.99 (83.62) | 592.59 (80.94) | 597.47 (89.87) | 583.56 (100.74) | 598.47 (84.38) | 588.57 (88.84) | 589.56 (111.67) | 592.20 (97.24) | 591.76 (83.92) |
|  | Alerting Index | 48.47 (23.22) | 69.11 (36.03) | 79.97 (28.72) | 40.73 (33.86) | 58.15 (36.33) | 62.26 (45.73) | 48.19 (31.66) | 62.49 (39.86) | 70.60 (35.65) | 60.00 (25.92) |
| Orienting Cue | Central Cue | 604.43 (87.10) | 620.90 (97.87) | 624.08 (92.31) | 610.13 (89.85) | 606.12 (105.87) | 625.63 (80.43) | 604.05 (95.24) | 612.79 (119.42) | 625.19 (112.32) | 614.81 (90.21) |
|  | Spatial Cue | 568.21 (85.13) | 572.53 (92.58) | 576.85 (88.66) | 588.04 (90.54) | 562.96 (94.08) | 586.60 (84.05) | 574.71 (95.25) | 573.38 (114.59) | 585.52 (102.34) | 576.53 (88.46) |
|  | Orienting Index | 36.22 (25.18) | 48.37 (31.19) | 47.23 (32.27) | 22.09 (23.34) | 43.15 (39.89) | 39.03 (38.36) | 29.34 (25.92) | 39.41 (24.94) | 39.68 (31.89) | 38.28 (22.40) |

**Appendix 2: Mean ERP Amplitudes Averaged across Stage of Task Performance**

**Table A2.1.**

ERP amplitudes (μV) with congruent and incongruent flankers in three groups (HG=Healthy group, SG = Subsyndromal group, DG = Depressed group).

| ERP | Electrode Site | Congruent | | | Incongruent | | | Executive Control Index | | |
| --- | --- | --- | --- | --- | --- | --- | --- | --- | --- | --- |
|  |  | HG | SG | DG | HG | SG | DG | HG | SG | DG |
| N100_tar | Fz | -.42 (1.8) | -.86 (1.2) | -.63 (1.1) | -.31 (1.8) | -.72 (1.1) | -.72 (1.2) | -.11 (1.0) | .13 (0.7) | -.09 (0.7) |
|  | (CPz+Pz)/2 | -1.08 (1.6) | -1.09 (1.4) | -1.09 (1.2) | -1.27 (0.6) | -.97 (1.2) | -.96 (1.2) | -.19 (0.8) | .13 (0.9) | -.14 (0.6) |
| P300_tar | Fz | 4.25 (1.7) | 2.45 (1.7) | 1.96 (1.1) | 4.04 (1.8) | 2.28 (1.8) | 2.03 (1.2) | .21 (1.1) | .17 (0.8) | -.07 (0.6) |
|  | (CPz+Pz)/2 | 3.92 (2.0) | 2.83 (1.8) | 2.29 (0.8) | 3.58 (2.0) | 2.47 (1.4) | 2.50 (0.8) | .34 (1.0) | .36 (1.0) | -.20 (0.5) |

**Table A2.2.**

ERP amplitudes (μV) at three groups in no cue and double cue conditions (HG=Healthy group, SG = Subsyndromal group, DG = Depressed group).

| ERPs | Electrode Site | No cue | | | Double cue | | | Alerting Index | | |
| --- | --- | --- | --- | --- | --- | --- | --- | --- | --- | --- |
|  |  | HG | SG | DG | HG | SG | DG | HG | SG | DG |
| N100_cue | Fz | -.75 (0.7) | -.82 (0.6) | -.61 (0.8) | -.72 (1.5) | -.81 (1.2) | -.61 (0.8) | .03 (1.5) | .01 (1.2) | -.01 (1.0) |
|  | (Pz+P3+P4+O1+O2)/5 | -1.05 (0.5) | -1.18 (0.8) | -1.16 (0.6) | -2.06 (1.1) | -1.92 (1.3) | -1.19 (0.7) | -1.01 (1.2) | -.74 (1.4) | -.04 (1.0) |
| N100_tar | Fz | -.93 (1.6) | -.83 (1.1) | -.34 (0.7) | -.70 (2.1) | -1.44 (1.7) | -1.21 (2.3) | .24 (1.5) | .61 (1.9) | -.87 (2.2) |
|  | (Pz+P3+P4+O1+O2)/5 | -2.39 (1.7) | -1.80 (1.5) | -1.45 (1.2) | -3.26 (1.9) | -2.73 (2.6) | -1.72 (1.8) | -.87 (1.2) | -.93 (1.5) | -.27 (1.7) |
| P300 | Fz | 3.78 (1.9) | 2.17 (1.4) | 2.30 (1.1) | 4.09 (1.9) | 2.63 (2.1) | 2.20 (1.3) | .31 (1.5) | .47 (1.5) | -.10 (1.3) |
|  | (Pz+P3+P4+O1+O2)/5 | 3.79 (1.2) | 2.65 (1.3) | 2.36 (0.9) | 3.89 (1.5) | 3.42 (1.6) | 3.18 (1.3) | .09 (1.2) | .78 (1.1) | .81 (1.7) |

**Table A2.3.**

ERP amplitudes (μV) at three groups in center cue and spatial cue conditions (HG=Healthy group, SG = Subsyndromal group, DG = Depressed group).

| ERPs | Electrode Site | No cue | | | Double cue | | | Alerting Index | | |
| --- | --- | --- | --- | --- | --- | --- | --- | --- | --- | --- |
|  |  | HG | SG | DG | HG | SG | DG | HG | SG | DG |
| N100_cue | Fz | -.95 (1.2) | -1.02 (0.9) | -.69 (0.9) | -1.20 (1.5) | -.83 (0.8) | -.49 (.07) | -.25 (1.1) | .19 (1.0) | .20 (0.9) |
|  | (Pz+P3+P4+O1+O2)/5 | -1.57 (0.9) | -1.39 (0.8) | -1.13 (0.8) | -1.77 (0.9) | -1.51 (1.0) | -1.17 (0.7) | -.20 (0.8) | -.12 (1.0) | -.03 (0.8) |
| N100_tar | Fz | -.16 (1.6) | -.96 (1.3) | -.59 (1.5) | -.23 (2.0) | -.81 (1.3) | -.70 (1.2) | -.08 (1.3) | -.14 (1.1) | -.11 (1.1) |
|  | (Pz+P3+P4+O1+O2)/5 | -2.88 (1.9) | -2.25 (2.1) | -1.53 (1.5) | -3.10 (1.7) | -2.28 (2.5) | -1.29 (1.6) | -.22 (1.0) | -.02 (1.1) | -.24 (1.0) |
| P300 | Fz | 4.39 (1.7) | 2.81 (1.6) | 2.20 (1.3) | 4.66 (2.0) | 2.86 (2.2) | 2.27 (1.2) | .27 (1.2) | -.05 (1.1) | .07 (0.9) |
|  | (Pz+P3+P4+O1+O2)/5 | 3.83 (1.5) | 3.11 (1.5) | 2.70 (0.9) | 3.61 (1.4) | 3.32 (1.5) | 3.08 (1.1) | -.22 (0.9) | .21 (0.8) | .12 (0.9) |

**Appendix 3: Mean ERP Amplitudes at each Stage of Task Performance**

**Table A3.1.**

Mean ERP amplitudes (μV) with congruent and incongruent flankers in three groups by stage of task performance (1^st^, 2^nd^, 3^rd^) (HG=Healthy group, SG = Subsyndromal group, DG = Depressed group).

| ERPs | Electrode Sites | Congruent | | | | | | | | | Incongruent | | | | | | | | |
| --- | --- | --- | --- | --- | --- | --- | --- | --- | --- | --- | --- | --- | --- | --- | --- | --- | --- | --- | --- |
|  |  | HG | | | SG | | | DG | | | HG | | | SG | | | DG | | |
|  |  | 1st | 2st | 3rd | 1st | 2st | 3rd | 1st | 2st | 3rd | 1st | 2st | 3rd | 1st | 2st | 3rd | 1st | 2st | 3rd |
| N100_tar | Fz | -.63 (2.3) | -.19 (2.0) | -.44 (1.8) | -1.18 (1.4) | -.60 (1.5) | -.79 (1.5) | -.84 (1.6) | -.52 (1.7) | -.52 (1.4) | -.37 (2.3) | -.26 (1.8) | -.30 (1.7) | -.99 (1.6) | -.60 (1.5) | -.57 (1.4) | -.39 (1.2) | -.77 (1.5) | -.99 (2.0) |
|  | (CPz+Pz)/2 | -1.51 (1.8) | -.81 (2.2) | -.92 (1.7) | -1.51 (2.1) | -.79 (1.3) | -.98 (1.6) | -1.27 (1.6) | -1.20 (1.4) | -.82 (1.4) | -1.83 (1.6) | -1.10 (1.9) | -.89 (1.8) | -1.17 (2.2) | -.72 (1.4) | -1.01 (1.6) | -.94 (1.7) | -1.03 (1.6) | -.91 (1.5) |
| P300_tar | Fz | 4.32 (1.8) | 4.28 (2.4) | 4.16 (1.9) | 2.38 (1.9) | 2.59 (2.0) | 2.38 (1.7) | 1.97 (1.3) | 1.97 (1.7) | 1.95 (1.3) | 3.80 (1.8) | 4.25 (2.3) | 4.08 (2.0) | 2.16 (2.1) | 2.39 (2.0) | 2.29 (1.8) | 2.39 (1.5) | 1.85 (1.5) | 1.86 (1.7) |
|  | (CPz+Pz)/2 | 4.01 (2.2) | 4.05 (2.4) | 3.69 (2.0) | 2.87 (2.1) | 3.04 (2.3) | 2.59 (1.4) | 2.66 (1.2) | 1.82 (1.2) | 2.40 (1.2) | 3.65 (2.1) | 3.59 (2.4) | 3.51 (1.9) | 2.54 (1.6) | 2.48 (1.7) | 2.39 (1.6) | 2.76 (1.2) | 2.24 (1.3) | 2.48 (1.1) |
| ERPs | Electrode Sites | Executive Control Index | | | | | | | | |  |  |  |  |  |  |  |  |  |
|  |  | HG | | | SG | | | DG | | |  |  |  |  |  |  |  |  |  |
|  |  | 1st | 2nd | 3rd | 1st | 2nd | 3rd | 1st | 2nd | 3rd |  |  |  |  |  |  |  |  |  |
| N100_tar | Fz | -.25 (1.5) | .07 (1.7) | -.14 (1.5) | -.18 (1.4) | .003 (1.5) | -.22 (1.5) | -.45 (1.4) | .24 (1.4) | .47 (1.7) |  |  |  |  |  |  |  |  |  |
|  | (CPz+Pz)/2 | .32 (1.1) | .30 (1.1) | -.03 (1.9) | -.34 (1.4) | -.07 (1.4) | .02 (1.4) | -.33 (1.2) | -.17 (1.1) | .09 (1.3) |  |  |  |  |  |  |  |  |  |
| P300_tar | Fz | .52 (1.9) | .03 (1.7) | .08 (1.7) | .22 (1.3) | .21 (1.5) | .09 (1.2) | -.42 (.87) | .12 (1.4) | .09 (1.4) |  |  |  |  |  |  |  |  |  |
|  | (CPz+Pz)/2 | .37 (1.4) | .46 (1.3) | .18 (1.4) | .33 (1.3) | .56 (1.6) | .19 (1.2) | -.10 (1.1) | -.42 (1.3) | -.08 (1.4) |  |  |  |  |  |  |  |  |  |

**Table A3.2.**

Mean ERP amplitudes (μV) with no cue and double cue in three groups by stage of task performance (1^st^, 2^nd^, 3^rd^) (HG=Healthy group, SG = Subsyndromal group, DG = Depressed group).

| ERPs | Electrode Sites | No cue | | | | | | | | | Double cue | | | | | | | | |
| --- | --- | --- | --- | --- | --- | --- | --- | --- | --- | --- | --- | --- | --- | --- | --- | --- | --- | --- | --- |
|  |  | HG | | | SG | | | DG | | | HG | | | SG | | | DG | | |
|  |  | 1st | 2nd | 3rd | 1st | 2nd | 3rd | 1st | 2nd | 3rd | 1st | 2nd | 3rd | 1st | 2nd | 3rd | 1st | 2nd | 3rd |
| N100_cue | Fz | -.42 (1.3) | -.82 (0.6) | -1.01 (1.1) | -.80 (0.9) | -.65 (1.0) | -1.00 (1.0) | -.51 (1.2) | -.57 (1.0) | -.74 (1.3) | -.68 (2.3) | -.91 (1.6) | -.56 (1.6) | -.94 (1.3) | -.94 (1.7) | -.55 (1.4) | -.83 (1.3) | -.53 (1.3) | -.46 (0.8) |
|  | (Pz+P3+P4+O1+O2)/5 | -.94 (0.9) | -1.03 (0.6) | -1.19 (0.90 | -.95 (0.9) | -1.32 (1.8) | -1.26 (1.1) | -1.35 (0.9) | -.99 (0.9) | -1.13 (0.8) | -2.43 (1.6) | -1.93 (1.4) | -1.83 (1.0) | -1.89 (1.8) | -2.03 (1.6) | -1.84 (1.3) | -1.39 (1.0) | -1.05 (1.2) | -1.14 (0.9) |
| N100_tar | Fz | -1.08 (2.4) | -.73 (1.6) | -.99 (1.8) | -.58 (1.1) | -.78 (1.4) | -1.14 (1.9) | -.48 (1.1) | -.35 (1.0) | -.20 (1.1) | -1.12 (2.8) | -.41 (2.3) | -.56 (2.1) | -1.95 (2.1) | -1.60 (2.1) | -.77 (2.1) | -1.18 (2.4) | -.95 (2.9) | -1.51 (2.9) |
|  | (Pz+P3+P4+O1+O2)/5 | -2.73 (1.9) | -2.25 (1.8) | -2.19 (2.0) | -1.80 (2.0) | -1.93 (1.8) | -1.67 (1.8) | -1.63 (1.6) | -1.41 (1.5) | -1.31 (1.3) | -3.78 (2.4) | -3.13 (2.4) | -2.87 (1.7) | -3.04 (3.1) | -2.69 (2.8) | -2.45 (2.5) | -1.93 (2.2) | -1.48 (2.0) | -1.75 (2.4) |
| P300_tar | Fz | 4.36 (2.4) | 3.64 (2.2) | 3.36 (1.8) | 2.25 (2.0) | 2.25 (1.4) | 2.01 (1.4) | 2.46 (1.6) | 2.25 (1.4) | 2.19 (1.4) | 4.10 (2.1) | 4.04 (2.3) | 4.13 (2.1) | 2.40 (2.1) | 2.55 (2.7) | 2.95 (2.2) | 2.67 (1.8) | 2.04 (1.7) | 1.88 (1.7) |
|  | (Pz+P3+P4+O1+O2)/5 | 4.31 (1.5) | 3.55 (1.4) | 3.53 (1.2) | 2.73 (1.5) | 2.64 (1.5) | 2.58 (1.5) | 2.57 (1.4) | 2.20 (1.0) | 2.33 (1.0) | 4.04 (1.9) | 3.72 (1.7) | 3.90 (1.5) | 3.50 (1.8) | 3.61 (2.5) | 3.17 (1.5) | 3.56 (1.7) | 3.13 (1.6) | 2.85 (1.4) |
| ERPs | Electrode Sites | Alerting Index | | | | | | | | |  |  |  |  |  |  |  |  |  |
|  |  | HG | | | SG | | | DG | | |  |  |  |  |  |  |  |  |  |
|  |  | 1st | 2nd | 3rd | 1st | 2nd | 3rd | 1st | 2nd | 3rd |  |  |  |  |  |  |  |  |  |
| N100_cue | Fz | -.26 (2.5) | -.09 (1.6) | .45 (2.0) | -.13 (1.5) | -.29 (2.1) | .45 (1.5) | -.33 (1.5) | .03 (1.5) | .29 (1.5) |  |  |  |  |  |  |  |  |  |
|  | (Pz+P3+P4+O1+O2)/5 | -1.48 (1.7) | -.91 (1.4) | -.65 (1.2) | -.94 (1.9) | -.71 (2.0) | -.58 (1.5) | -.04 (1.2) | -.06 (1.6) | -.00 (1.3) |  |  |  |  |  |  |  |  |  |
| N100_tar | Fz | -.04 (1.8) | .32 (1.9) | .43 (2.1) | -1.37 (2.2) | -.83 (2.3) | .38 (2.8) | -.71 (2.5) | -.59 (2.5) | -1.31 (2.9) |  |  |  |  |  |  |  |  |  |
|  | (Pz+P3+P4+O1+O2)/5 | -1.04 (1.8) | -.88 (1.5) | -.68 (1.7) | -1.24 (1.8) | -.76 (1.9) | -.78 (2.5) | -.30 (2.0) | -.07 (1.9) | -.43 (2.2) |  |  |  |  |  |  |  |  |  |
| P300_tar | Fz | -.26 (1.8) | .40 (2.5) | .77 (1.8) | .15 (2.1) | .30 (2.0) | .94 (2.2) | .21 (2.5) | -.21 (1.4) | -.31 (1.4) |  |  |  |  |  |  |  |  |  |
|  | (Pz+P3+P4+O1+O2)/5 | -.27 (1.5) | .18 (1.5) | .37 (1.5) | .77 (1.7) | .97 (2.1) | .59 (1.6) | .99 (2.4) | .93 (1.8) | .52 (1.9) |  |  |  |  |  |  |  |  |  |

**Table A3.3.**

Mean ERP amplitudes (μV) with central and spatial cue in three groups by stage of task performance (1^st^, 2^nd^, 3^rd^) (HG=Healthy group, SG = Subsyndromal group, DG = Depressed group).

| ERPs | Electrode Sites | Central cue | | | | | | | | | Spatial cue | | | | | | | | |
| --- | --- | --- | --- | --- | --- | --- | --- | --- | --- | --- | --- | --- | --- | --- | --- | --- | --- | --- | --- |
|  |  | HG | | | SG | | | DG | | | HG | | | SG | | | DG | | |
|  |  | 1st | 2nd | 3rd | 1st | 2nd | 3rd | 1st | 2nd | 3rd | 1st | 2nd | 3rd | 1st | 2nd | 3rd | 1st | 2nd | 3rd |
| N100_cue | Fz | -1.16 (1.5) | -.80 (1.8) | -.90 (1.2) | -1.05 (1.0) | -1.07 (1.1) | -.92 (1.5) | -.82 (1.2) | -.68 (1.3) | -.58 (1.2) | -1.21 (1.9) | -1.18 (1.9) | -1.22 (1.7) | -.64 (1.0) | -.61 (1.5) | -1.23 (1.3) | -.53 (1.1) | -.39 (0.9) | -.55 (1.0) |
|  | (Pz+P3+P4+O1+O2)/5 | -1.83 (1.3) | -1.49 (1.2) | -1.39 (1.1) | -1.49 (0.9) | -1.32 (1.3) | -1.36 (1.2) | -1.38 (1.0) | -.95 (0.9) | -1.07 (1.5) | -1.9 (1.1) | -1.6 (1.0) | -1.84 (1.2) | -1.54 (1.4) | -1.41 (1.2) | -1.6 (1.3) | -1.3 (0.9) | -1.09 (1.0) | -1.12 (1.0) |
| N100_tar | Fz | -.22 (1.8) | -.29 (1.9) | .04 (1.9) | -1.24 (1.8) | -1.00 (1.7) | -.62 (1.7) | -.58 (2.2) | -.82 (1.8) | -.38 (1.5) | -.60 (2.6) | -.02 (2.1) | -.08 (2.7) | -.90 (1.9) | -.57 91.4) | -.97 (2.0) | -.67 (1.6) | -.75 (1.7) | -.69 (1.5) |
|  | (Pz+P3+P4+O1+O2)/5 | -3.48 (2.3) | -2.66 (1.9) | -2.49 (2.0) | -2.72 (2.7) | -1.85 (2.3) | -2.19 (1.8) | -1.55 (1.7) | -1.58 (2.0) | -1.45 (1.9) | -3.69 (1.9) | -2.69 (2.0) | -2.92 (1.9) | -2.50 (2.9) | -2.17 (2.5) | -2.18 (2.6) | -1.20 (2.1) | -1.36 (1.9) | -1.29 (1.6) |
| P300_tar | Fz | 4.19 (1.8) | 4.45 (2.5) | 4.52 (2.0) | 2.75 (1.8) | 2.88 (1.9) | 2.81 (2.1) | 2.45 (1.6) | 2.07 (1.6) | 2.07 (1.4) | 4.41 (2.1) | 5.05 (2.8) | 4.52 (2.2) | 3.00 (2.2) | 3.13 (2.8) | 2.46 (2.2) | 2.34 (1.5) | 2.28 (1.7) | 2.18 (1.7) |
|  | (Pz+P3+P4+O1+O2)/5 | 3.83 (1.8) | 3.91 (1.9) | 3.75 (1.3) | 3.19 (1.6) | 3.19 (1.7) | 2.94 (1.8) | 2.81 (1.2) | 2.67 (1.5) | 2.61 (1.1) | 3.59 (1.7) | 3.88 (1.8) | 3.36 (1.4) | 3.49 (1.5) | 3.44 (1.9) | 3.02 (1.9) | 3.30 (1.3) | 3.07 (1.4) | 2.86 (1.6) |
| ERPs | Electrode Sites | Orienting Index | | | | | | | | |  |  |  |  |  |  |  |  |  |
|  |  | HG | | | SG | | | DG | | |  |  |  |  |  |  |  |  |  |
|  |  | 1st | 2nd | 3rd | 1st | 2nd | 3rd | 1st | 2nd | 3rd |  |  |  |  |  |  |  |  |  |
| N100_cue | Fz | -.05 (1.6) | -.39 (1.7) | -.33 (1.5) | .42 (1.1) | .46 (2.0) | -.30 (1.7) | .29 (1.5) | .29 (1.3) | .03 (1.3) |  |  |  |  |  |  |  |  |  |
|  | (Pz+P3+P4+O1+O2)/5 | -.02 (1.0) | -.12 (1.4) | -.46 (1.1) | -.05 (1.2) | -.09 (1.5) | -.23 (1.5) | .09 (1.3) | -.14 (0.8) | -.05 (1.6) |  |  |  |  |  |  |  |  |  |
| N100_tar | Fz | -.39 (2.1) | .27 (1.9) | -.12 (2.6) | .34 (1.6) | .43 (1.8) | -.34 (1.8) | -.09 (2.3) | .07 (1.6) | -.31 (1.2) |  |  |  |  |  |  |  |  |  |
|  | (Pz+P3+P4+O1+O2)/5 | -.21 (1.6) | -.03 (1.2) | -.44 (1.5) | .22 (1.2) | -.32 (1.6) | .01 (2.1) | .35 (1.5) | .22 (2.3) | .16 (1.6) |  |  |  |  |  |  |  |  |  |
| P300_tar | Fz | .22 (1.6) | .60 (2.2) | .005 (2.0) | .26 (1.5) | .25 (2.1) | -.35 (1.6) | -.11 (1.3) | .21 (2.0) | .11 (1.4) |  |  |  |  |  |  |  |  |  |
|  | (Pz+P3+P4+O1+O2)/5 | -.24 (1.7) | -.03 (1.5) | -.39 (1.2) | .32 (1.2) | .25 (1.1) | .08 (2.2) | .49 (1.2) | .40 (1.7) | .25 (1.3) |  |  |  |  |  |  |  |  |  |
